# Infarct volume versus NIHSS score for ischemic and bleeding risk stratification after acute ischemic stroke

**DOI:** 10.1016/j.mmr.2026.100067

**Published:** 2026-09-16

**Authors:** Zi-Mo Chen, Ying-Yu Jiang, Hong-Qiu Gu, Xin-Miao Zhang, Jing Jing, Jing-Lin Mo, Yong Jiang, Li-Ping Liu, Xing-Quan Zhao, Yong-Jun Wang, Zi-Xiao Li

**Affiliations:** a Department of Neurology, Beijing Tiantan Hospital, Capital Medical University, Beijing 100071, China; b China National Clinical Research Center for Neurological Diseases, Beijing Tiantan Hospital, Capital Medical University, Beijing 100071, China; c National Center for Healthcare Quality Management in Neurological Diseases, Beijing Tiantan Hospital, Capital Medical University, Beijing 100071, China; d Advanced Innovation Center for Human Brain Protection, Capital Medical University, Beijing 100071, China; e Research Unit of Artificial Intelligence in Cerebrovascular Disease, Chinese Academy of Medical Sciences, Beijing 100071, China; f Center for Excellence in Brain Science and Intelligence Technology, Chinese Academy of Sciences, Shanghai 200031, China

**Keywords:** Ischemic stroke (IS), Infarct volume, National Institutes of Health Stroke Scale (NIHSS), Stroke recurrence, Intracerebral hemorrhage (ICH)

## Abstract

**Background:**

Whether infarct volume refines assessment of recurrent ischemic stroke (IS) and intracerebral hemorrhage (ICH) risk following acute IS beyond the National Institutes of Health Stroke Scale (NIHSS) remains uncertain. We compared their performance in ischemic-hemorrhagic risk stratification.

**Methods:**

We included 10,545 patients with acute IS from the Third China National Stroke Registry (CNSR-III) with diffusion-weighted imaging/apparent diffusion coefficient-derived infarct volume and NIHSS data. Outcomes were 90-day recurrent IS and ICH, analyzed separately. Two reciprocal Cox models were used: one with infarct volume adjusted for NIHSS, and another with NIHSS adjusted for infarct volume. Stratified analyses, restricted cubic splines, and grouped threshold analyses were exploratory. In the GOLDEN BRIDGE II study, recurrent IS and all bleeding events were assessed for external consistency.

**Results:**

In CNSR-III, larger infarct volume was independently associated with recurrent IS [hazard ratio (*HR*)=1.35, 95% confidence interval (CI) 1.25–1.47] and ICH (*HR*=2.48, 95% CI 2.11–2.91) after NIHSS adjustment, whereas NIHSS lost significance after infarct-volume adjustment (IS: *HR*=1.05, 95% CI 0.89–1.24; ICH: *HR*=1.11, 95% CI 0.78–1.58). ICH risk rose around 9.0 ml, with increased hazard at 9.0–9.9 ml (*HR*=3.61, 95% CI 1.29–10.07), while IS risk increased without a sharp rise (*HR*=1.15, 95% CI 0.98–1.35). Patients with NIHSS score ≥4 and infarct volume <9.0 ml had IS risk comparable to NIHSS score ≤3 but potentially lower ICH risk (*HR*=0.63, 95% CI 0.38–1.02). Infarct volume 9.0–17.9 ml increased ICH risk (*HR*=1.96, 95% CI 1.06–3.63). Infarct volume was <9.0 ml in 66.7% of NIHSS score 6–15 and 25.4% of NIHSS score ≥16. GOLDEN BRIDGE II confirmed principal findings, with infarct volume associated with recurrent IS (*HR*=1.37, 95% CI 1.25–1.49) and bleeding events (*HR*=1.53, 95% CI 1.31–1.78).

**Conclusions:**

Infarct volume better characterizes post-stroke ischemic-hemorrhagic risk than NIHSS. Prospective validation is required before using infarct volume to guide antiplatelet selection.

## 1 Background

Stroke is the leading cause of death and disability in China. In 2020, an estimated 17.8 million adults had experienced stroke, 3.4 million had a first-ever stroke, and 2.3 million died from stroke, while 12.5% of stroke survivors were left disabled [1]. Risk stratification after acute ischemic stroke (IS) requires profiling the competing hazards of recurrent ischemia and intracerebral hemorrhage (ICH) so that early secondary prevention can be contextualized within an individualized ischemic-hemorrhagic risk framework [2], [3]. In current practice, the National Institutes of Health Stroke Scale (NIHSS) is the most widely applied clinical gauge of initial neurological deficit and is frequently used when anticipating hemorrhagic complications and guiding early management.

Short-course dual antiplatelet therapy has demonstrated well-established efficacy in selected patients with minor stroke or high-risk transient ischemic attack; accordingly, many contemporary trial frameworks and guidance documents evaluate “stroke severity” using NIHSS thresholds (e.g., NIHSS ≤3 or ≤5) when contextualizing early secondary prevention [4], [5], [6], [7], [8], [9]. However, despite its widespread use, NIHSS has important limitations in capturing lesion burden and biological injury. Patients with moderate-to-severe stroke generally have worse prognoses and more harmful consequences from recurrence than those with minor stroke [10], [11], [12], [13], [14], raising the question of whether NIHSS-based eligibility alone adequately captures the relevant pathobiological profile for balancing ischemic benefit against bleeding risk, and highlighting the potential need for imaging-derived measures such as infarct volume.

Conceptually, whereas NIHSS quantifies the neurological impairment assessed clinically, infarct volume quantifies the imaging-derived estimate of infarcted tissue. The two measures quantify different aspects of stroke severity, and they may stratify post-stroke ischemic and hemorrhagic risks differently [15], [16]. Despite this, their comparative performance for reflecting the early ischemic-hemorrhagic risk profile after acute IS remains insufficiently characterized.

In this study, we aimed to compare infarct volume and NIHSS in reflecting the 90-day ischemic-hemorrhagic risk profile after acute IS and to assess external support for the infarct volume-risk associations in GOLDEN BRIDGE II. We also performed exploratory analyses to evaluate whether an infarct-volume-based operational small-infarct boundary (<9.0 ml) provided additional ischemic-hemorrhagic risk stratification beyond the conventional “minor stroke” definition (NIHSS ≤3).

## 2 Methods

### 2.1 Study cohort and participants

This study analyzed data from the prospective nationwide Third China National Stroke Registry study, which was conducted between August 2015 and March 2018 and included 15,166 patients with acute IS from 201 hospitals across China [17]. Eligible participants were aged 18 or older and enrolled within 7 d of experiencing acute symptoms of IS. The study was approved by the Beijing Tiantan Hospital institutional review board (KY2015-001-01) and adhered to the principles of the Declaration of Helsinki. Written informed consent was obtained from all study participants or their representatives. Our current analysis focused specifically on patients diagnosed with acute IS who had data available for the NIHSS score on admission and for the infarction volume measured on diffusion weighted imaging (DWI) and apparent diffusion coefficient (ADC) images.

### 2.2 External validation analyses in GOLDEN BRIDGE II

To assess external consistency, we used data from GOLDEN BRIDGE II (ClinicalTrials.gov identifier: NCT04524624; approval number: KY 2020-016-02), a recently established multicenter cluster-randomized clinical trial evaluating a clinical decision support system in acute ischemic stroke [18]. The validation analysis included 14,949 patients with available baseline NIHSS and infarct-volume data. Recurrent IS and all bleeding events within 90 d were assessed in GOLDEN BRIDGE II. Because hemorrhagic transformation was not systematically ascertained in GOLDEN BRIDGE II, the CNSR-III definition of ICH could not be reproduced. Therefore, all bleeding events were used to evaluate the hemorrhagic-risk dimension in the external consistency analyses.

### 2.3 Data collection and variables of interest

Baseline information included patient demographics (age and sex), body mass index (BMI), history of disease (smoking status, hypertension, diabetes mellitus, disorders of lipid metabolism, IS, and myocardial infarction), previous medication history [antihypertensive, hypoglycemic, and lipid-lowering drugs, intravenous thrombolytic therapy (recombinant tissue-type plasminogen activator or urokinase)], medication at discharge (antiplatelet and anticoagulant agents), and time interval between stroke onset and MRI scan.

The NIHSS score was calculated during direct interviews conducted by trained research coordinators at the time of admission. To evaluate the significance of increasing NIHSS score and infarct volume, we defined an elevated NIHSS score as the cohort median or higher and assessed increased infarct volume as per the standard deviation of logarithmically transformed (base e) (ln-transformed) values [19], to facilitate comparison of effect estimates across variables measured on different scales; clinically relevant NIHSS categories were further evaluated in stratified analyses. We then assessed their impact on the overall population and across stratified analyses according to NIHSS score and infarct volume categories.

Neurologists used the TOAST (Trial of ORG 10172 in Acute Stroke Treatment) classification to determine the causes of stroke [20]. Stroke etiologies were centrally classified by the Tiantan Neuroimaging Center into five categories: large-artery atherosclerosis, cardioembolism, small-vessel occlusion, stroke of other determined etiology, and stroke of undetermined etiology [21]. The data were interpreted by two experienced neurologists. Any disagreements were resolved by consultation with a third senior neurologist.

Recurrent IS and ICH within 90 d were analyzed as separate outcomes representing the ischemic and hemorrhagic dimensions of the post-stroke risk profile after acute IS. In this study, ICH referred to imaging-confirmed intracranial bleeding during follow-up, including hemorrhagic transformation and primary intracerebral hemorrhage, as confirmed by computed tomography or magnetic resonance imaging (MRI). Hemorrhagic transformation, classified according to the European Cooperative Acute Stroke Study [22], involves bleeding into an area previously affected by ischemia, whereas primary intracerebral hemorrhage refers to spontaneous bleeding within the brain parenchyma not initially associated with IS.

### 2.4 Measurement of infarct volume

Infarct volume was measured on DWI and ADC images using an automated *U*-Net (*U*-shaped convolutional neural network) segmentation model previously developed in CNSR-III. The model generated lesion masks from preprocessed axial DWI images with a *b*-value of 1000 s/mm², and corresponding ADC images, and infarct volume was calculated from the segmented lesion voxels. The model achieved a median Dice score of 0.910 [interquartile range (IQR) 0.839–0.935], with detailed development, training, validation, and performance metrics described previously [23].

NIHSS and infarct-volume categorizations were selected according to the analytic purpose. In the primary reciprocal models, NIHSS was dichotomized at the cohort-specific median (<4 vs. ≥4 in CNSR-III; <3 vs. ≥3 in GOLDEN BRIDGE II), whereas infarct volume was modeled per SD of its ln-transformed value because of its right-skewed distribution.

Clinically oriented exploratory analyses used NIHSS score ≤3 to represent conventional minor stroke, with additional categories of ≤3, 4–5, 6–15, and ≥16 used to characterize increasing neurological deficit. Within each NIHSS category, infarct volume was modeled per standard deviation (SD) of its ln-transformed value, with further adjustment for continuous NIHSS to account for residual within-category variation.

Conversely, NIHSS associations were examined within infarct-volume strata of <5.0, 5.0–9.9, 10.0–29.9, 30.0–69.9, and ≥70.0 ml. These boundaries were broadly aligned with volume ranges used in previous stroke-imaging studies, in which 5 and 10 ml have been used to distinguish smaller-volume lesions and 30 and 70 ml to characterize moderate-to-large ischemic cores. These strata were informed by published volumetric schemes using boundaries at 5, 10, 20, 30, 40, and 50 ml, as well as the 70-ml ischemic-core threshold used in imaging-selected stroke trials [24], [25]. Adjacent published ranges were combined and adapted to the distribution of infarct volume in the present cohort to retain adequate patients and outcome events. Within each infarct-volume stratum, NIHSS was dichotomized at the stratum-specific median, resulting in cut points of <3, <4, <5, <6, and <11, respectively, with further adjustment for continuous ln-transformed infarct volume.

**Fig. 1:**
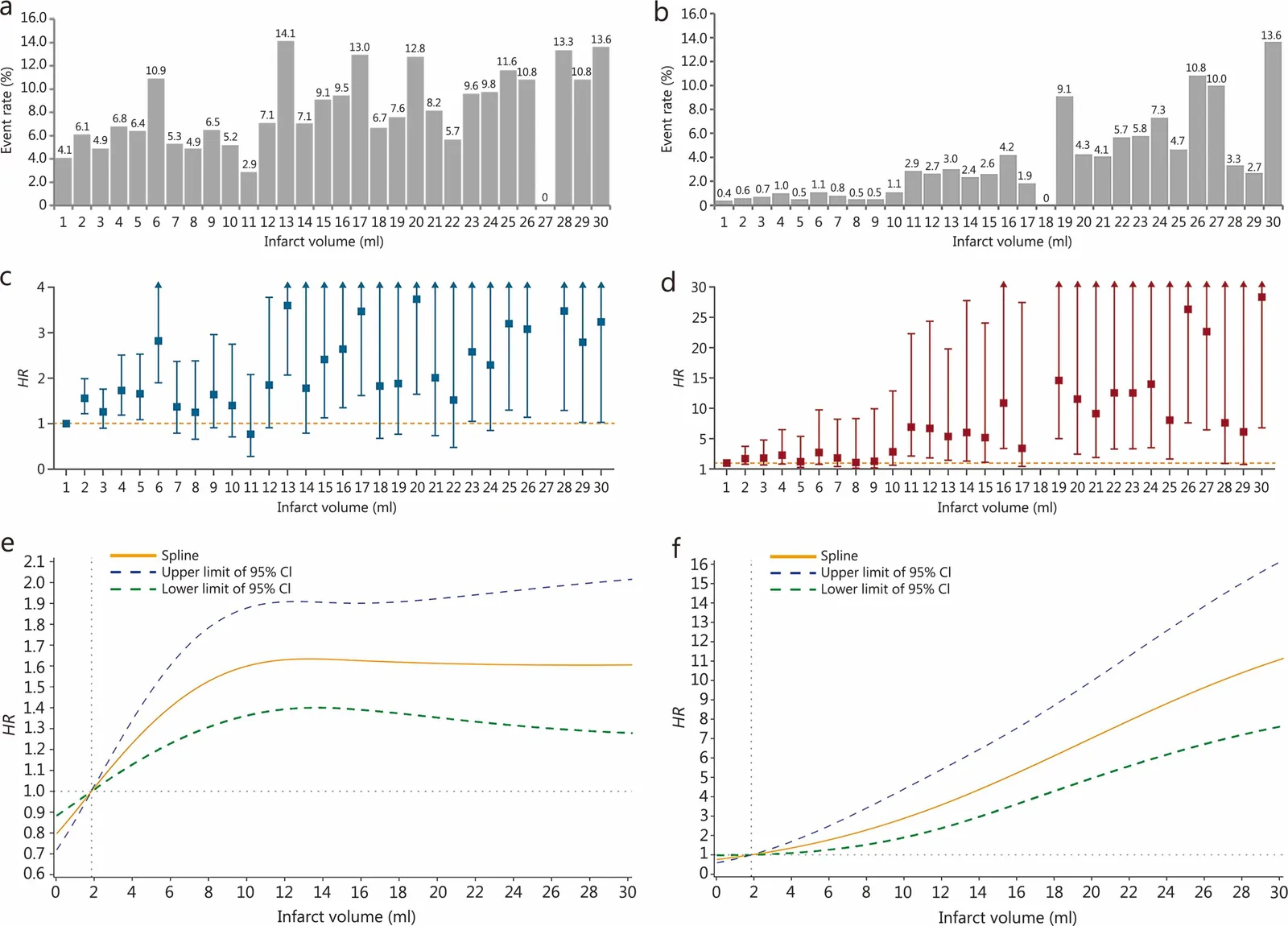
Exploratory dose-response patterns of infarct volume with recurrent ischemic stroke and intracerebral hemorrhage in CNSR-III. Event rates for recurrent ischemic stroke **(a)** and intracerebral hemorrhage **(b)** according to 1-ml infarct-volume intervals. The vertical red dotted line in panel b indicates the beginning of the transition-like increase in intracerebral hemorrhage risk. Adjusted hazard ratios (*HR*s) and 95% confidence intervals (CIs) for recurrent ischemic stroke **(c)** and intracerebral hemorrhage **(d)** across 1-ml infarct-volume intervals. Restricted cubic spline curves showing the continuous associations of infarct volume with recurrent ischemic stroke **(e)** and intracerebral hemorrhage **(f)**. Orange line indicates *HR* estimate; blue dotted line indicates 95% CI; vertical dotted line indicates median infarct volume (reference, *HR*=1). Models adjusted for age, sex, body mass index, medical history (smoking, hypertension, diabetes, disorders of lipid metabolism, ischemic stroke, and myocardial infarction), medication history (antihypertensives, hypoglycemics, lipid-lowering agents), intravenous thrombolysis, discharge antiplatelet, and anticoagulant therapy.

The 9.0-ml operational boundary was derived from the exploratory risk-pattern analyses presented in “Patterns of infarct volume on risks of IS and ICH” section and Fig. 1. The resulting <9.0, 9.0–17.9, and ≥18.0 ml categories, and the finer 3-ml and 1-ml intervals, were used only for exploratory risk-pattern characterization and were not considered validated clinical cutoffs. Additional analyses were stratified by sex, age (<65 years vs. ≥65 years), and TOAST etiology. For circulation-specific correlation analyses, patients with infarcts involving both the anterior and posterior circulations were excluded from the anterior-only and posterior-only subgroups but retained in the total-population analysis. Other infarct-topology analyses were treated as exploratory sensitivity analyses, as described in “Sensitivity analysis” section.

### 2.5 Mediation analysis

We performed mediation analysis to assess the influence of the NIHSS score on the association between infarct volume and study outcomes utilizing a counterfactual framework. The details are presented in **Additional file 1: Materials and methods**.

### 2.6 Sensitivity analysis

Several prespecified sensitivity and exploratory analyses were performed to assess the robustness and consistency of the principal findings. These included additional adjustments for TOAST etiology and the NIHSS-MRI time interval, stratification by the time from stroke onset to MRI, separate analyses of hemorrhagic transformation and primary intracerebral hemorrhage, analyses accounting for infarct topology, extended follow-up at 1 and 5 years, sequential exclusion cohorts, and Fine-Gray competing-risk models treating death as a competing event. For location-specific analyses, 728 patients with both anterior- and posterior-circulation involvement were excluded from the location-specific subgroups but retained in the total-population analysis.

### 2.7 Statistical analysis

Dichotomous variables are presented as the proportion and continuous variables as the mean±standard deviation (SD) or median (IQR). Continuous and categorical variables were compared between groups using the Wilcoxon rank-sum and chi-squared tests. Associations of infarct volume and the NIHSS score with IS and ICH were analyzed using multivariate Cox regression models, which yielded *HR*s with 95% CIs. Baseline information covariates, which were selected based on a review of the literature [26], [27] and clinical experience, were adjusted in the multivariate Cox regression models and mediation analyses. Two complementary Cox model frameworks were prespecified: ln(infarct volume) as the exposure (adjusted for NIHSS and prespecified covariates) and, reciprocally, NIHSS as the exposure [adjusted for ln(infarct volume) and the same covariates]; the exposure of interest was not included again as an adjustment covariate.

The analyses included evaluation of infarct volume on a continuous scale using restricted cubic spline (RCS) curves, with the median infarct volume as the reference. The correlation between infarct volume and the NIHSS score was examined using scatter plots and the Spearman rank method. Bootstrap resampling was used to enhance the stability of the results. The details are provided in the **Additional file 1: Materials and methods**. Harrell’s C-index was calculated as an ancillary direct model-comparison measure for base clinical models, base plus NIHSS, base plus infarct volume, and base plus both. Multiplicity adjustment was not applied to exploratory and supportive analyses; therefore, subgroup, mediation, spline, TOAST, topology, bootstrap, grouped-threshold, and sensitivity analyses were considered exploratory and supportive and were interpreted cautiously without confirmatory claims.

All statistical analyses were performed using SAS version 9.4 (SAS Institute Inc., Cary, NC, USA). All *P*-values were two-sided and considered statistically significant at *P*<0.05.

## 3 Results

### 3.1 Baseline characteristics of the patients

The primary CNSR-III analysis included 10,545 patients with acute IS, among whom 631 experienced recurrent IS and 184 developed ICH within 90 d. The study flow diagram is shown in **Additional file 1:** Fig. S1. The demographic and clinical characteristics of the study population categorized by infarct volume are shown in **Additional file 1:** Table S1. The average patient age was (62.3±11.2) years, and 30.5% were female. The baseline characteristics of the included and excluded patients are shown in **Additional file 1:** Table S2. Compared with excluded patients, included patients were less often female (30.5% vs. 34.2%) and more often previous or current smokers (32.5% vs. 28.7%); most other baseline differences were small. The GOLDEN BRIDGE II external validation cohort included 14,949 patients, with 504 recurrent IS events and 154 all-bleeding events within 90 d; baseline characteristics are shown in **Additional file 1:** Table S3.

### 3.2 Associations of infarct volume and NIHSS score with IS and ICH

Even after adjusting for the NIHSS score, a larger infarct volume remained significantly associated with increased risks of recurrent IS (*HR*=1.35, 95% CI 1.25–1.47, *P*<0.001) and ICH (*HR*=2.48, 95% CI 2.11–2.91, *P*<0.001) **(**Table 1**)**. Furthermore, stratified analysis confirmed that within restricted NIHSS score ranges, larger infarct volumes were significantly associated with increased risks of both recurrent IS and ICH **(Additional file 1:** Table S4**)**.

**Table 1:** Associations of infarct volume and NIHSS score with recurrent ischemic stroke (IS) and intracerebral hemorrhage (ICH).

**Variable**	**Events/Patients (%)**	**Unadjusted model**				**Adjusted model**^⁎^				**Adjusted mode**^**l**†/‡^	
		***HR*****(95% CI)**	***P*****-value**			***HR*****(95% CI)**	***P*****-value**			***HR*****(95% CI)**	***P*****-value**
Recurrent IS											
Infarct volume											
Per SD of ln-transformed infarct volume	631/10,545 (5.98)	1.34 (1.24−1.44)	<0.001			1.33 (1.24−1.44)	<0.001			1.35 (1.25−1.47)^†^	<0.001^†^
NIHSS score											
NIHSS score <4 (reference)	275/5235 (5.25)	1	-			1	-			1	-
NIHSS score ≥4	356/5310 (6.70)	1.29 (1.10−1.51)	0.002			1.21 (1.03−1.42)	0.022			1.05 (0.89−1.24)^‡^	0.578^‡^
ICH											
Infarct volume											
Per SD of ln-transformed infarct volume	184/10,545 (1.74)	3.13 (2.73−3.59)	<0.001			2.62 (2.27−3.03)	<0.001			2.48 (2.11−2.91)^†^	<0.001^†^
NIHSS score											
NIHSS score <4 (reference)	51/5235 (0.97)	1	-			1	-			1	-
NIHSS score ≥4	133/5310 (2.50)	2.64 (1.91−3.66)	<0.001			2.01 (1.44−2.81)	<0.001			1.11 (0.78–1.58)^**‡**^	0.560^**‡**^

Standard deviation (SD) values of ln-transformed infarct volume were calculated, with an SD of 1.61, and the median NIHSS score was 4. “-” indicates not applicable. ^⁎^Adjusted for age, sex, body mass index, medical history (smoking, history of hypertension, diabetes mellitus, disorders of lipid metabolism, ischemic stroke, myocardial infarction), medication history (antihypertensive, hypoglycemic, and lipid-lowering drugs, intravenous thrombolytic therapy), medication at discharge (antiplatelet and anticoagulant drugs). ^†^Adjusted for the variables in ^⁎^ plus NIHSS score. ^‡^Adjusted for the variables in ^⁎^ plus standardized ln-transformed infarct volume. CI. Confidence interval; *HR*. Hazard ratio; ln. Logarithmically transformed (base e); NIHSS. National Institutes of Health Stroke Scale

A higher NIHSS score was associated with significantly increased risks of IS (*HR*=1.21, 95% CI 1.03–1.42, *P*=0.022) and ICH (*HR*=2.01, 95% CI 1.44–2.81, *P*<0.001) after adjusting for baseline covariates excluding infarct volume **(**Table 1**)**. However, when infarct volume was included as a covariate in adjustments, the associations between a higher NIHSS score and both IS (*HR*=1.05, 95% CI 0.89–1.24, *P*=0.578) and ICH (*HR*=1.11, 95% CI 0.78–1.58, *P*=0.560) were no longer significant, indicating that the initial findings were confounded by infarct volume. Furthermore, the stratified analysis confirmed that within restricted infarct volume ranges, higher NIHSS scores were not associated with an increased risk of IS or ICH **(Additional file 1:** Table S5**)**. The associations remained without meaningful change when analyses were stratified by age (<65 years vs. ≥65 years) and by sex, as detailed in **Additional file 1:** Tables S6-S9, with the same adjustment as the main analyses.

**Additional file 1:** Fig. S2 shows that the NIHSS score did not mediate the association between infarct volume and an increased risk of recurrent IS (*PM=*–5.27%, 95% CI –16.42 to 5.88, *P*=0.354) or ICH (*PM=*5.21%, 95% CI –0.29 to 10.71, *P*=0.063).

Ancillary Harrell’s C-index analyses provided direct model-comparison metrics **(Additional file 1:** Table S10**)**. In CNSR-III, adding infarct volume to the base clinical model improved discrimination more than adding NIHSS for recurrent IS (*HR*=0.61 vs. 0.57; *P*=0.001) and ICH (*HR*=0.81 vs. 0.71; *P*<0.001).

### 3.3 Patterns of infarct volume on risks of IS and ICH

Fig. 1**a, c** show that the event rates and *HR*s for recurrent IS increased with infarct volume without a sharp rise; in model-based quantitative analyses, all HRs were estimated per 1-unit increment in ln-transformed infarct volume. Infarct volume was associated with recurrent IS < 9.0 ml (*HR*=1.27, 95% CI 1.15–1.39, *P*<0.001) but not at ≥9.0 ml (*HR*=1.15, 95% CI 0.98–1.35, *P*=0.095). Conversely, Fig. 1**b, d** show that ICH risk remained relatively stable < 9.0 ml (*HR*=1.19, 95% CI 0.89–1.58, *P*=0.243) but increased significantly at ≥9.0 ml (*HR*=1.61, 95% CI 1.35–1.91, *P*<0.001).

The RCS analysis revealed that the risk of IS increased with infarct volume until it reached 12.0–13.0 ml, at which point the upward trend gradually stabilized (Fig. 1**e; Additional file 1:** Fig. S3a). In contrast, the rising trend of ICH risk continued until the infarct volume reached approximately 40 ml, at which point it gradually stabilized (Fig. 1**f; Additional file 1:** Fig. S3b). These findings illustrate different exploratory dose-response patterns for recurrent IS and ICH with increasing infarct volume.

### 3.4 Exploratory comparison of infarct volume <9.0 ml versus an NIHSS score ≤3 in refinement of ischemic-hemorrhagic risk

**Fig. 2:**
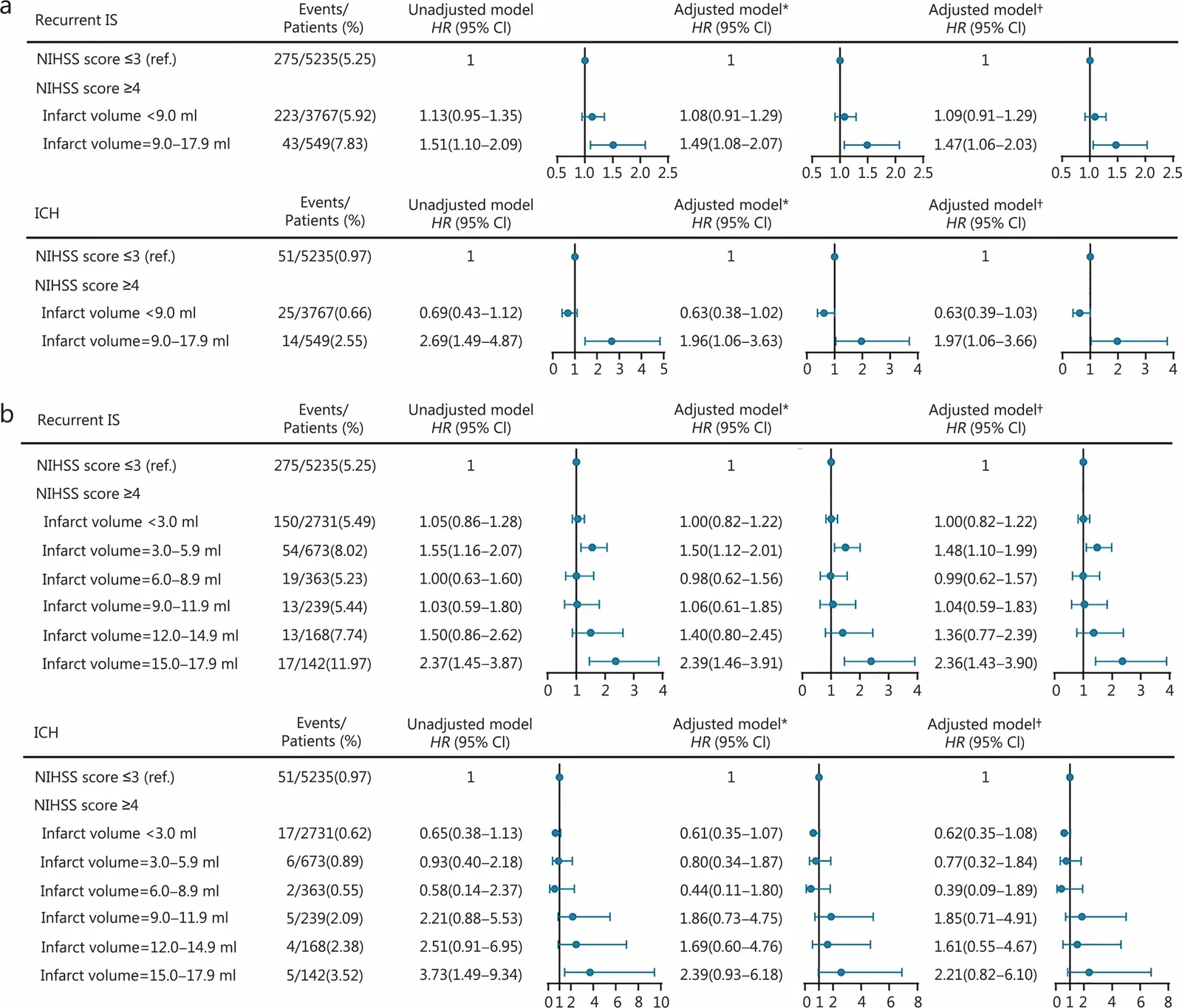
Comparison of the risk of recurrent IS and intracerebral hemorrhage between “infarct volumes <9.0 ml” and “NIHSS score ≤3”. ^⁎^Adjusted for age, sex, body mass index, medical history (smoking, history of hypertension, diabetes mellitus, disorders of lipid metabolism, ischemic stroke, myocardial infarction), medication history (antihypertensive, hypoglycemic, and lipid-lowering drugs, intravenous thrombolytic therapy), and discharge antiplatelet and anticoagulant therapy. ^†^The bootstrap-corrected *HR* and 95% CI were based on 1000 independent bootstrap runs. Both subfigures **a** and **b** include data from 9551 patients. *HR*. Hazard ratio; CI. Confidence interval; NIHSS. National Institutes of Health Stroke Scale; ref. Reference; IS. Ischemic stroke; ICH. Intracerebral hemorrhage.

Fig. 2**a** demonstrates that, compared to patients with NIHSS score ≤3, those with higher NIHSS score ≥4 but smaller infarct volumes <9.0 ml had a comparable risk of recurrent IS (*HR*=1.08, 95% CI 0.91–1.29, *P*=0.385) but a potentially lower risk of ICH (*HR*=0.63, 95% CI 0.38–1.02, *P*=0.061). However, when an infarct volume reached the range of 9.0–17.9 ml, the risks of IS and ICH were significantly increased (recurrent IS: *HR*=1.49, 95% CI 1.08–2.07, *P*=0.015; ICH: *HR*=1.96, 95% CI 1.06–3.63, *P*=0.033). This comparison was used to characterize the risk profile of patients with imaging-defined small infarcts, rather than to establish <9.0 ml as a definitive clinical cutoff.

In detail, compared to patients with an NIHSS score ≤3, among those with an NIHSS score >3, all stratified subgroups of infarct volumes <9.0 ml exhibited comparable or increased risks of IS (<3.0 ml: *HR*=1.00, 95% CI 0.82–1.22; 3.0–5.9 ml: *HR*=1.50, 95% CI 1.12–2.01; 6.0–8.9 ml: *HR*=0.98, 95% CI 0.62–1.56). Similarly, all the three subgroups showed consistent trends towards lower risk of ICH (<3.0 ml: *HR*=0.61, 95% CI 0.35–1.07; 3.0–5.9 ml: *HR*=0.80, 95% CI 0.34–1.87; 6.0–8.9 ml: *HR*=0.44, 95% CI 0.11–1.80) (Fig. 2**b**). Compared to patients with an NIHSS score ≤3, among those with an NIHSS score >3, all stratified subgroups of infarct volumes 9.0–17.9 ml exhibited consistent trends towards higher risk of ICH (9.0–11.9 ml: *HR*=1.86, 95% CI 0.73–4.75; 12.0–14.9 ml: *HR*=1.69, 95% CI 0.60–4.76; 15.0–17.9 ml: *HR*=2.39, 95% CI 0.93–6.18). The findings were similar for IS (9.0–11.9 ml: *HR*=1.06, 95% CI 0.61–1.85; 12.0–14.9 ml: *HR*=1.40, 95% CI 0.80–2.45; 15.0–17.9 ml: *HR*=2.39, 95% CI 1.46–3.91).

### 3.5 Exploratory consistency analyses of infarct volume <9.0 ml versus an NIHSS score ≤3

Among patients classified as having minor stroke (NIHSS score ≤3), the risks of IS and ICH were higher in patients with an infarct volume of ≥9.0 ml than in those with an infarct volume of <9.0 ml (*HR*=1.85, 95% CI 1.39–2.46, *P*<0.001 and *HR*=8.55, 95% CI 4.76–15.23, *P*<0.001, respectively) **(Additional file 1:** Fig. S4a**)**. Therefore, the clinical implications of an NIHSS score ≤3 when characterizing the risks of IS and ICH still varied according to infarct volume.

In contrast, among patients with an infarct volume of <9.0 ml, there was no significant difference in the risk of IS or ICH according to whether the NIHSS score was ≤3, 4–5, or ≥6 **(Additional file 1:** Fig. S4b**)**.

### 3.6 TOAST-stratified patterns of infarct volume on risks of IS and ICH

For the TOAST-stratified RCS analyses restricted to infarct volumes <70 ml, 343 patients with infarct volume ≥70 ml were excluded. The plotted population included 2706 patients with large-artery atherosclerosis, 604 with cardioembolism, 2690 with small-artery occlusion, 135 with other determined etiology, and 4067 with undetermined etiology. These counts were derived from the CNSR-III analytic dataset after exclusion of patients with infarct volume ≥70 ml.

TOAST-stratified RCS analyses suggested that the positive relationship between infarct volume and recurrent IS was most apparent in etiologies directly or potentially related to vasculopathy [28], including large-artery atherosclerosis, small-artery occlusion, and stroke of undetermined etiology, whereas the infarct volume-ICH curves showed heterogeneous but generally upward trends, with wide confidence intervals in smaller etiologic subgroups **(Additional file 1:** Figs. S5, S6**)**.

### 3.7 Sensitivity analysis

Results were materially consistent after additional adjustment for TOAST etiology and the NIHSS-MRI time interval, and after stratification by onset-to-MRI time **(Additional file 1:** Tables S11-S13**)**. After further adjustment for TOAST etiology and the time interval between NIHSS assessment and MRI scan, the associations of infarct volume and NIHSS score with recurrent IS and ICH remained consistent with the primary analyses **(Additional file 1:** Table S11**)**. Similar findings were observed after stratification by onset-to-MRI time (0–2 d vs. ≥3 d): NIHSS score was not significantly associated with the risk of recurrent IS or ICH, whereas infarct volume remained significantly associated with both outcomes in each time-interval subgroup **(Additional file 1:** Tables S12, S13**)**.

Sensitivity analyses of hemorrhagic subtype, infarct topology, extended follow-up, and robustness re-analyses yielded broadly consistent infarct volume-bleeding associations **(Additional file 1:** Tables S14-S25**)**. When hemorrhagic transformation and primary ICH were analyzed separately, the infarct volume-hemorrhagic outcome associations remained broadly consistent with those observed for the overall ICH outcome in the primary analysis **(Additional file 1:** Tables S14, S15**)**. In analyses accounting for infarct topology, anterior-circulation involvement was the only topology feature independently associated with recurrent IS after adjustment for infarct volume, whereas none of the evaluated topology features showed an independent association with ICH **(Additional file 1:** Tables S16-S18**)**. Across extended follow-up analyses, sequential exclusion cohorts, and Fine-Gray competing-risk models treating death as a competing event, effect estimates were materially consistent with the primary 90-day Cox analyses **(Additional file 1:** Tables S19-S25**)**.

### 3.8 Discordance between infarct volume and the NIHSS score

**Fig. 3:**
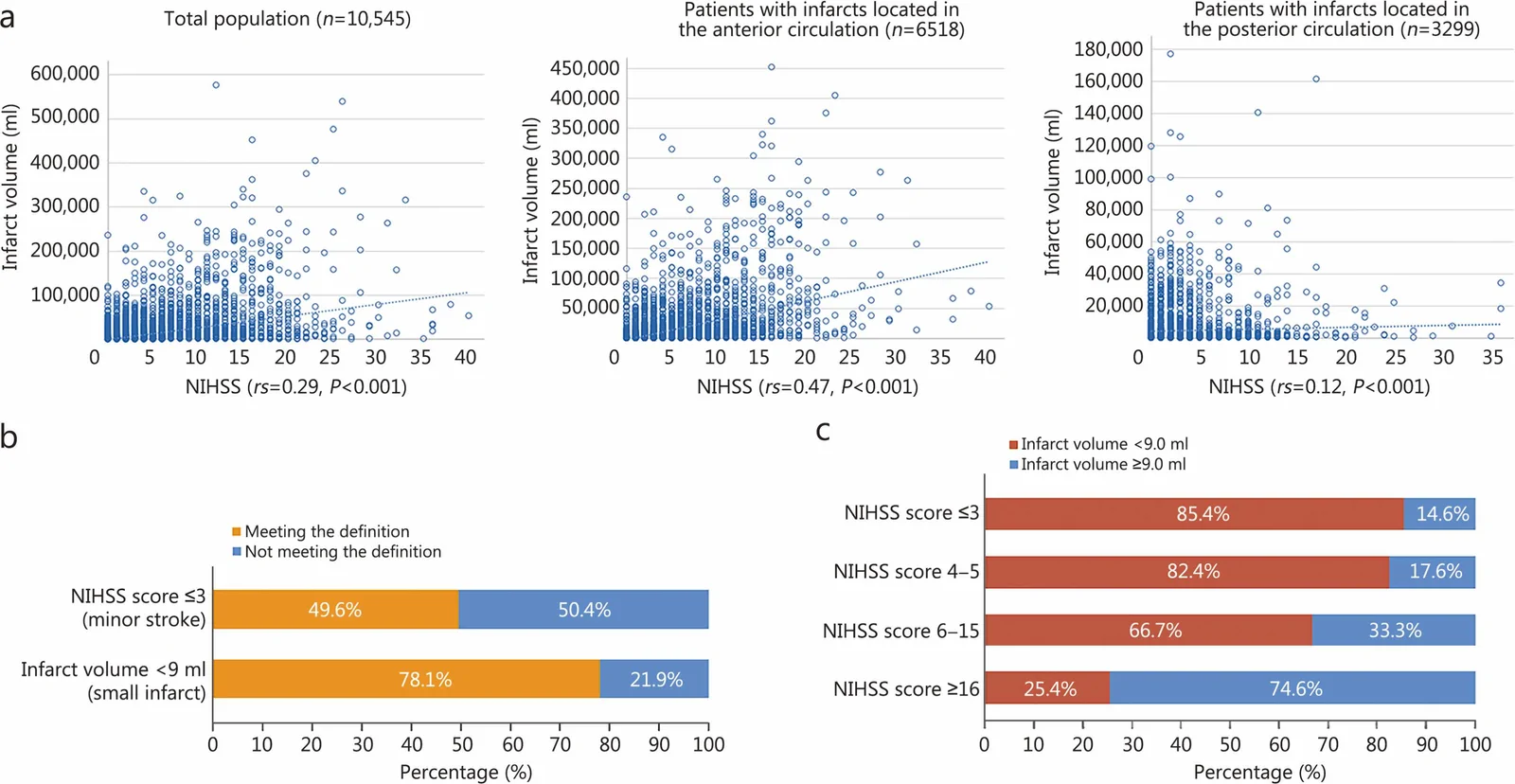
Correlation between infarct volume and the national institutes of health stroke scale (NIHSS) Score. **a** Correlation between infarct volume and NIHSS score in total population (*n*=10,545). Correlation between infarct volume and NIHSS score in patients with infarcts located in the anterior circulation (*n*=6518). Correlation between infarct volume and NIHSS score in patients with infarcts located in the posterior circulation (*n*=3299). **b** Prevalence of the total population meeting the NIHSS score ≤3 criterion (5235/10,545) and the infarct volume <9.0 ml criterion (8236/10,545). **c** Prevalen**c**e of infarct volume <9.0 ml within prespecified NIHSS subgroups: NIHSS score ≤3 (*n*=5235), 4−5 (*n*=2130), 6−15 (*n*=2916), and ≥16 (*n*=264). The <9.0 ml bou*n*dary was used as an exploratory operational definition of imaging-defined small infarcts and was not intended to establish a definitive clinical cutoff. NIHSS. National Institutes of Health Stroke Scale.

Fig. 3**a** shows that the overall correlation between infarct volume and the NIHSS score was relatively weak (*rs*=0.29, *P*<0.001). A stronger correlation was noted for infarcts located in the anterior circulation (*rs*=0.47, *P*<0.001) than for those in the posterior circulation (*rs*=0.12, *P*<0.001) (Fig. 3**a**). A larger proportion of patients had infarct volumes <9.0 ml than NIHSS score ≤3 in the total population (78.1% vs. 49.6%) (Fig. 3**b**). When stratified by NIHSS score, 85.4% of patients with an NIHSS score ≤3 and 82.4% of patients with an NIHSS score of 4–5 had an infarct volume of <9.0 ml. Notably, even among patients categorized as having moderate stroke (NIHSS score 6–15) and severe stroke (NIHSS score ≥16), a substantial proportion (66.7% and 25.4%, respectively) still had an infarct volume <9.0 ml (Fig. 3**c**).

### 3.9 External validation in GOLDEN BRIDGE II

In GOLDEN BRIDGE II, infarct volume remained independently associated with recurrent IS (adjusted *HR* per SD of ln-transformed infarct volume=1.37, 95% CI 1.25–1.49, *P*<0.001) and all bleeding events (adjusted *HR*=1.53, 95% CI 1.31–1.78, *P*<0.001) after adjustment for clinical covariates and NIHSS **(**Table 2**)**. Conversely, NIHSS was not independently associated with recurrent IS (adjusted *HR*=1.15, 95% CI 0.96–1.38, *P*=0.127) or all bleeding events (adjusted *HR*=0.80, 95% CI 0.57–1.12, *P*=0.198) after adjustment for clinical covariates and infarct volume **(**Table 2**)**. In ancillary discrimination analyses, infarct volume improved discrimination for recurrent IS compared with NIHSS (C-index 0.61 vs. 0.57, *P*=0.002), whereas the difference for all bleeding events was directionally consistent but not statistically significant (C-index 0.65 vs. 0.63, *P*=0.245) **(Additional file 1:** Table S10**)**. Models including both NIHSS and infarct volume yielded C-indices of 0.62 for recurrent IS and 0.66 for all bleeding events.

**Table 2:** External validation of infarct volume and NIHSS score for 90-day outcomes in GOLDEN BRIDGE II.

**Variable**	**Events/ Patients (%)**	**Unadjusted model**		**Adjusted model**^**⁎**^		**Adjusted model**^**†/‡**^	
		***HR*****(95% CI)**	***P*****-value**	***HR*****(95% CI)**	***P*****-value**	***HR*****(95% CI)**	***P*****-value**
Recurrent IS							
Infarct volume							
Per SD of ln-transformed infarct volume	504/14,949 (3.37)	1.31 (1.21–1.42)	<0.001	1.31 (1.21–1.42)	<0.001	1.37 (1.25–1.49)^†^	<0.001^†^
NIHSS score							
NIHSS score <3 (ref.)	277/8178 (3.39)	1	-	1	-	1	-
NIHSS score ≥3	227/6771 (3.35)	0.99 (0.83–1.18)	0.878	1.04 (0.87–1.24)	0.667	1.15 (0.96–1.38)^‡^	0.127^‡^
All bleeding events							
Infarct volume							
Per SD of ln-transformed infarct volume	154/14,949 (1.03)	1.66 (1.43–1.91)	<0.001	1.65 (1.43–1.91)	<0.001	1.53 (1.31–1.78)^†^	<0.001^†^
NIHSS score							
NIHSS score <3 (ref.)	100/8178 (1.22)	1	-	1	-	1	-
NIHSS score ≥3	54/6771 (0.80)	0.65 (0.47–0.90)	0.010	0.67 (0.48–0.94)	0.019	0.80 (0.57–1.12)^‡^	0.198^‡^

The SD of ln-transformed infarct volume was calculated in GOLDEN BRIDGE II, and NIHSS was dichotomized at the cohort-specific median of 3. “-” indicates not applicable. ^⁎^Adjusted for age, sex, body mass index, and medical history, including smoking, hypertension, diabetes mellitus, disorders of lipid metabolism, ischemic stroke, and myocardial infarction. ^†^Adjusted for the variables in ^⁎^ plus NIHSS score. ^‡^Adjusted for the variables in ^⁎^ plus standardized ln-transformed infarct volume. CI. Confidence interval; *HR*. Hazard ratio; ln. Logarithmically transformed (base **e**); NIHSS. National Institutes of Health Stroke Scale; SD. Standard deviation; IS. Ischemic stroke.

Categorical analyses in GOLDEN BRIDGE II were directionally consistent with the CNSR-III findings **(Additional file 1:** Table S26**)**. Compared with infarct volume <9.0 ml, infarct volumes of 9.0–17.9 ml and ≥18.0 ml were associated with higher risks of recurrent IS (adjusted *HR*s 1.44 vs. 1.67) and all bleeding events (adjusted *HR*s 2.36 vs. 2.97) after adjustment for clinical covariates and NIHSS. Exploratory 1-ml interval analyses suggested a transition-like increase in the risk of all bleeding events around 9.0 ml, with the event rate rising from 0.65% in the <1.0 ml reference stratum to 2.40% in the 9.0–9.9 ml stratum (adjusted *HR*=3.61, 95% CI 1.29–10.07) and elevated estimates observed in several subsequent higher-volume strata. No comparable transition-like pattern was observed for recurrent IS in the 1-ml interval analyses, providing external supportive evidence consistent with the CNSR-III finding that the 9.0-ml operational boundary primarily characterized hemorrhagic-risk patterns rather than a recurrent-ischemic-risk threshold **(Additional file 1:** Tables S27, S28**)**. RCS curves showed increasing risks of recurrent IS and all bleeding events with larger infarct volume, with a steeper increase for bleeding; these curves were exploratory and were not used to define a clinical cutoff **(Additional file 1:** Fig. S7**)**.

## 4 Discussion

The findings of this study demonstrate that infarct volume appeared to better reflect the ischemic-hemorrhagic risk profile after acute IS than the NIHSS score. Notably, an infarct volume of <9.0 ml was associated with a comparable risk of recurrent IS but a lower risk of ICH in comparison with the “minor stroke” classification (NIHSS score ≤3), even in patients with higher NIHSS scores. Furthermore, an infarct-volume boundary of <9.0 ml identified substantially more patients than the conventional minor stroke category and revealed a sizeable subgroup with moderate-to-severe neurological deficits but relatively small infarcts. This boundary should be interpreted as an exploratory operational threshold rather than an established clinical cutoff.

The primary clinical implication is that, where infarct volume can be reliably calculated, it may be prioritized over the NIHSS for stratifying the competing risks of recurrent IS and ICH [13], [14], [29], [30], [31], [32]. Despite the higher recurrence risk among IS patients with moderate-to-high NIHSS scores, current guidelines largely reserve dual antiplatelet therapy for minor stroke/TIA [13], [14]. In our cohort, we observed a notable mismatch between NIHSS score and infarct volume, showing that a significant proportion of patients with moderate or severe stroke have relatively small infarcts (volume <9.0 ml). The weak correlation between NIHSS and infarct volume may partly reflect known limitations of NIHSS, including differential weighting of cortical, language, and posterior circulation deficits. Additionally, an “infarct volume <9.0 ml” criterion captures more IS patients compared to an “NIHSS score ≤3”. Accordingly, emphasizing imaging-defined small infarcts rather than the NIHSS-based minor-stroke label may refine selection of patients for future trials of intensified antiplatelet therapy, particularly among patients with moderate-to-severe clinical deficits, as reflected by NIHSS, but small infarcts. Prospective studies should refine volumetric cutoffs and confirm generalizability.

Our study revealed that the risk of ICH associated with increasing infarct volume follows an “initially flat, followed by a marked rise” pattern. Specifically, within an infarct volume of <9.0 ml, the risk of ICH remains relatively stable. Conversely, the risk of IS within this volume range continues to increase, but without such a surge. This finding underscores the determinant role of ICH in disrupting the ischemic-hemorrhagic balance when the infarct volume reaches approximately 9.0 ml. Supporting this, patients with infarct volumes <9.0 ml and higher NIHSS scores exhibited comparable IS risk but lower ICH risk than those with NIHSS score ≤3. This pattern supports further evaluation of <9.0 ml as an operational boundary for a more favorable ischemic-hemorrhagic risk profile, but not as a definitive clinical cutoff.

We found that the risk of recurrent IS plateaued at a lower infarct volume than did the risk of ICH (12 ml vs. 40 ml). Thus, infarct volume may be a more critical determinant of the risk of ICH than of the risk of IS. Exploratory analyses based on the TOAST classification provided potential mechanistic insights. They revealed that the increase in risk of IS is associated with vasculopathy-related etiologies, likely because of more severe luminal stenosis, a heavier plaque burden, or more vulnerable plaques leading to larger infarcts [32], [33], [34]. In contrast, the increase in risk of ICH appears to be independent of the TOAST classification, occurring across all etiologies. These exploratory findings suggest that a larger infarct burden may contribute more directly to hemorrhagic vulnerability, whereas the relationship between infarct volume and recurrent IS may be partly influenced by underlying vasculopathy. This interpretation is biologically plausible: larger cores entail broader peri-infarct tissue below critical perfusion thresholds, with greater microvascular and blood-brain barrier injury [35], together with clinical observations of secondary injury in hemorrhagic tissue [36]. These findings provide a plausible rationale for the transition-like rise in ICH risk at higher infarct volumes.

Although the RCS curve could not directly identify a precise infarct volume cutoff at which ICH risk significantly rises, the 1-ml interval analyses showed that ICH event rates and *HR*s began to increase around 9.0 ml. GOLDEN BRIDGE II provided support for a transition-like increase in bleeding risk around this range, but could not validate an exact 9-ml cutoff. Furthermore, patients with higher NIHSS scores but infarct volumes <9.0 ml demonstrated a potentially lower ICH risk compared to those with NIHSS score ≤3, whereas infarct volumes ≥9.0 ml corresponded to higher ICH risk.

Several features strengthen the interpretation of these findings, including a large sample size of more than 10,000 patients with IS and detailed data on infarct volume, NIHSS scores, and covariates, as well as external supportive evidence from GOLDEN BRIDGE II, all of which contributed to statistical robustness and allowed the use of sophisticated analytical models, thereby enhancing the reliability and depth of its findings.

## 5 Limitations

First, this was an observational analysis and was not designed to determine whether infarct-volume-guided antiplatelet selection improves clinical outcomes. Although we compared infarct volume and NIHSS as alternative severity constructs, the study was not intended to develop a new prediction model. Residual confounding and indication bias cannot be excluded, particularly regarding the timing of symptom onset, treatment, clinical assessment, and imaging. Therefore, the findings should be interpreted as risk-stratification evidence and hypothesis-generating support for future prospective studies.

Second, the <9.0 ml boundary was derived from CNSR-III risk-pattern analyses and should be regarded as an exploratory operational marker of imaging-defined small infarcts, not as a validated clinical cutoff. Multiplicity adjustment was not applied to exploratory subgroup, mediation, spline, TOAST, topology, or threshold-related analyses; these analyses were used to assess consistency and biological plausibility rather than to make confirmatory inferences.

Third, infarct volume was measured using MRI/DWI-based automated segmentation, which may limit applicability in settings where acute MRI is not readily available. Because DWI/ADC-derived infarct volume primarily reflects established cytotoxic injury at the time of imaging, timing variability between clinical assessment and MRI acquisition may influence volume estimation. We partly addressed this by adjusting for the NIHSS-MRI interval and by confirming lesions on ADC to reduce T2 shine-through; however, standardized early imaging and serial infarct-volume assessment would further strengthen future studies. Moreover, although the segmentation algorithm had been previously developed, this clinical study did not revalidate its technical performance across centers, scanners, or acquisition protocols. Future studies should assess technical generalizability and explore scalable infarct-volume estimation approaches, including CT-based or cross-modality methods [37].

Fourth, hemorrhagic outcome definitions differed between CNSR-III and GOLDEN BRIDGE II. In CNSR-III, ICH included primary intracerebral hemorrhage and hemorrhagic transformation; sensitivity analyses separating these components showed broadly consistent volume-bleeding associations. In GOLDEN BRIDGE II, hemorrhagic transformation was not systematically ascertained, so the CNSR-III ICH definition could not be reproduced; all bleeding events were therefore used as the available hemorrhagic outcome for external consistency analyses.

Fifth, generalizability may be limited because inclusion required available DWI/ADC-derived infarct volume and complete NIHSS data, and CNSR-III was a China-based registry. Validation in independent, multicenter, multiethnic cohorts is needed.

## 6 Conclusions

This study highlights the potential value of prioritizing infarct volume over the NIHSS score when assessing the risks of recurrent IS and ICH after acute IS. Reframing risk stratification from “minor stroke” to imaging-defined “small infarct” may help identify selected patients with moderate-to-severe clinical deficits but small infarcts for future trials of intensified antiplatelet strategies. Whether infarct-volume-guided antiplatelet selection improves clinical outcomes requires prospective interventional validation.

## Abbreviations

ADC: Apparent diffusion coefficient

BMI: Body mass index

CNSR-III: Third China National Stroke Registry

CI: Confidence interval

DWI: Diffusion-weighted imaging

*HR*: Hazard ratio

ICH: Intracerebral hemorrhage

IS: Ischemic stroke

IQR: Interquartile range

NIHSS: National Institutes of Health Stroke Scale

MRI: Magnetic resonance imaging

PM: Proportion mediated

RCS: Restricted cubic spline

SD: Standard deviation

TOAST: Trial of ORG 10172 in acute stroke treatment

## Ethics approval and consent to participate

The study was approved by the Beijing Tiantan Hospital institutional review board (KY2015-001-01) and adhered to the principles of the Declaration of Helsinki.

## Funding

This study was supported by the Ministry of Science and Technology of China (2022YFC2504900; 2022YFC2504902) in the study design and conduct of the study, and the Beijing Municipal Natural Science Foundation (Z200016) in the collection, management, analysis, and interpretation of the data.

## Data Availability

Not applicable.

## Footnotes

Appendix A Supplementary data associated with this article can be found in the online version at doi:10.1016/j.mmr.2026.100067.
