# Supplementary material for: Infarct volume versus NIHSS score for ischemic and bleeding risk stratification after acute ischemic stroke
Source: Mil Med Res. 2026 Sep 16;13(1):100067. doi: 10.1016/j.mmr.2026.100067 (PMC13597881; doi:10.1016/j.mmr.2026.100067)
Supplement: Supplementary file 1 [file mmc1.pdf]

Materials and methods

Mediation analysis

To clarify the mediation effect of NIHSS score in the relationship between infarct volume and study outcomes [ischemic stroke (IS) and intracerebral hemorrhage (ICH)], we performed a causal mediation analysis within a counterfactual framework [1]. This approach enabled us to distinguish the direct effect of infarct volume from its indirect effect mediated by National Institutes of Health Stroke Scale (NIHSS) (treated as a continuous variable). We partitioned the total effect (TE) of infarct volume on outcomes into two parts: the natural direct effect (NDE) and the natural indirect effect (NIE), expressed as odds ratios (*OR*s). The NDE represented the impact of infarct volume on outcomes independent of NIHSS, while the NIE reflected the portion mediated by changes in NIHSS. The mediation effect was evaluated using the percentage mediated (PM), calculated as the ratio of NIE to TE multiplied by 100%. This methodology involved fitting two logistic regression models: one for NIHSS score (mediator) conditional on infarct volume and confounders, and another for study outcomes conditional on both infarct volume and NIHSS score, adjusting for all relevant confounders, including patient demographic parameters (age, sex), BMI, history of disease (smoking status, hypertension, diabetes mellitus, lipid metabolism disorders, ischemic stroke, and myocardial infarction); medication history: antihypertensive drugs, hypoglycemic drugs, lipid-lowering drugs, intravenous thrombolytic therapy (with recombinant tissue-type plasminogen activator or urokinase); discharge medication: antiplatelet agents, anticoagulant agents. It adheres to A Guideline for Reporting Mediation Analysis of Randomized Trials and Observational Studies (AGReMA) Statement [2].

Bootstrap method

The bootstrap resampling method was employed to enhance the reliability of the findings due to relatively low incidence rates of the study outcomes after stratification [3]. This method involved generating 1000 samples from the original dataset to assess the stability and consistency of results. The aggregate results, including the mean and standard deviation of the *HR*s, were analyzed for robustness.

Sensitivity analysis

To account for the confounding effects of different stroke etiologies including atrial fibrillation, the potential impact of measuring NIHSS and infarct volume at different time points, and recanalization, we performed sensitivity analyses. These analyses further adjusted for TOAST etiologies and the time interval between NIHSS assessment and MRI scan.

Additionally, we stratified patients based on the time interval between stroke onset and MRI scan, dividing them at the cohort median of 2 d into two groups: 0–2 d and ≥3 d. Within these subgroups, we examined the significance of the NIHSS score and infarct volume in determining the study outcomes.

Moreover, we separated hemorrhagic transformation and primary hemorrhagic stroke events and analyzed their relationship with infarct volume (0–30 ml, with each 1 ml increment) in terms of hazard ratios (*HR*s) with 95% confidence intervals (CIs).

We also investigated whether other characteristics of infarct topology independently influenced the study outcomes after accounting for infarct volume. These analyses distinguished between cortical and non-cortical infarcts, single and multiple infarcts, and anterior and posterior circulation infarcts.

To assess temporal generalizability and robustness, we conducted further analyses in the following prespecified sequence: first, we repeated the primary Cox models at extended time horizons (1 year and 5 years post-stroke); second, we re-estimated the 90-day models in sequential exclusion cohorts, removing i) patients with a history of prior stroke, ii) those receiving pre-stroke antithrombotic therapy (antiplatelet or anticoagulant agents), iii) patients with prior cardiovascular disease (peripheral arterial disease, pulmonary embolism, or heart disease), hepatic or renal impairment, or pre-existing disability [modified Rankin Scale (mRS≥2)], and iv) patients who underwent reperfusion therapy (intravenous thrombolysis with tPA or urokinase, intra-arterial thrombolysis, mechanical thrombectomy, or stent placement); finally, recognizing death as a competing event for both recurrent IS and ICH, the main analysis were reanalyzed using Fine-Gray subdistribution hazard models.

References

1. Valeri L, VanderWeele TJ. Mediation analysis allowing for exposure-mediator interactions and causal interpretation: theoretical assumptions and implementation with SAS and SPSS macros. Psychol Methods. 2013;18(2):137-50. https://doi.org/10.1037/a0031034
2. Lee H, Cashin AG, Lamb SE, Hopewell S, Vansteelandt S, VanderWeele TJ, *et al*. A guideline for reporting mediation analyses of randomized trials and observational studies: the AGReMA statement. JAMA. 2021;326(11):1045-56. https://doi.org/10.1001/jama.2021.14075.
3. Carpenter J, Bithell J. Bootstrap confidence intervals: when, which, what? A practical guide for medical statisticians. Stat Med. 2000;19(9):1141-64. https://doi.org/10.1002/(sici)1097-0258(20000515)19:9<1141::aid-sim479>3.0.co;2-f.

Table S1 Baseline characteristics of the patients grouped according to different infarct volumes

Characteristics	Total patients (*n*=10,545)	Infarct volume					*P*-value
		**< 5.0 ml (*n*=7308)**	**5.0–9.9 ml (*n*=1102)**	**10.0–29.9 ml (*n*=1239)**	**30.0–69.9 ml (*n*=553)**	**≥70.0 ml (*n*=343)**	
Age (year, mean±SD)	62.3±11.2	62.3±11.0	62.4±11.8	62.2±11.8	63.0±11.9	62.2±11.9	0.499
Female [*n* (%)]	3221 (30.5)	2279 (31.2)	350 (31.8)	347 (28.0)	147 (26.6)	98 (28.6)	0.031
BMI [kg/m^2, median (IQR)]	24.5 (22.6–26.6)	24.6 (22.8–26.7)	24.4 (22.6–26.3)	24.2 (22.1–26.3)	24.0 (22.1–26.4)	23.8 (21.9–25.4)	<0.001
Previous or current smoking [*n* (%)]	3424 (32.5)	2346 (32.1)	361 (32.8)	425 (34.3)	180 (32.5)	112 (32.7)	0.663
History of disease [*n* (%)]							
Hypertension	6657 (63.1)	4797 (65.6)	677 (61.4)	706 (57.0)	303 (54.8)	174 (50.7)	<0.001
Diabetes mellitus	2461 (23.3)	1833 (25.1)	229 (20.8)	221 (17.8)	106 (19.2)	72 (21.0)	<0.001
Lipid metabolism disorders	785 (7.4)	569 (7.8)	80 (7.3)	85 (6.9)	36 (6.5)	15 (4.4)	0.121
IS	2116 (20.1)	1496 (20.5)	230 (20.9)	221 (17.8)	104 (18.8)	65 (19.0)	0.211
Myocardial infarction	183 (1.7)	112 (1.5)	18 (1.6)	27 (2.2)	12 (2.2)	14 (4.1)	0.005
Previous medication [*n* (%)]							
Antihypertensive drugs	4705 (44.6)	3374 (46.2)	462 (41.9)	508 (41.0)	227 (41.0)	134 (39.1)	<0.001
Hypoglycemic drugs	1940 (18.4)	1459 (20.0)	172 (15.6)	185 (14.9)	73 (13.2)	51 (14.9)	<0.001
Lipid-lowering drugs	1051 (10.0)	725 (9.9)	95 (8.6)	119 (9.6)	64 (11.6)	48 (14.0)	0.037
Intravenous thrombolytic therapy	912 (8.6)	518 (7.1)	105 (9.5)	153 (12.3)	73 (13.2)	63 (18.4)	<0.001
Discharge medication [*n* (%)]							
Antiplatelet drugs	9579 (90.8)	6842 (93.6)	991 (89.9)	1050 (84.7)	454 (82.1)	242 (70.6)	<0.001
Anticoagulant drugs	305 (2.9)	112 (1.5)	50 (4.5)	80 (6.5)	40 (7.2)	23 (6.7)	<0.001
Time interval between stroke onset and MRI scan [h, median (IQR)]	2.0 (1.0–4.0)	2.0 (1.0–4.0)	2.0 (1.0–4.0)	2.0 (1.0–4.0)	3.0 (1.0–4.0)	2.0 (1.0–5.0)	<0.001

SD. Standard deviation; BMI. Body mass index; IQR. Interquartile range; MRI. Magnetic resonance imaging; IS. Ischemic stroke

Table S2 Baseline characteristics of the included and excluded patients

**Characteristics**	**Total patients (*n*=15,166)**	**Included population (*n*=10,545)**	**Excluded population (*n*=4621)**	***P*-value**
Age (year, mean±SD)	62.2±11.3	62.3±11.2	62.0±11.5	0.181
Female [*n* (%)]	4802 (31.7)	3221 (30.5)	1581 (34.2)	<0.001
BMI [kg/m^2, median (IQR)]	24.5 (22.6–26.6)	24.5 (22.6–26.6)	24.5 (22.6–26.4)	0.804
Previous or current smoking [*n* (%)]	4752 (31.3)	3424 (32.5)	1328 (28.7)	<0.001
History of disease [*n* (%)]				
Hypertension	9494 (62.6)	6657 (63.1)	2837 (61.4)	0.042
Diabetes mellitus	3510 (23.1)	2461 (23.3)	1049 (22.7)	0.392
Lipid metabolism disorders	1191 (7.9)	785 (7.4)	406 (8.8)	0.005
IS	3149 (20.8)	2116 (20.1)	1033 (22.4)	0.001
Myocardial infarction	292 (1.9)	183 (1.7)	109 (2.4)	0.010
Previous medication [*n* (%)]				
Antihypertensive drugs	6795 (44.8)	4705 (44.6)	2090 (45.2)	0.487
Hypoglycemic drugs	2773 (18.3)	1940 (18.4)	833 (18.0)	0.587
Lipid-lowering drugs	1671 (11.0)	1051 (10.0)	620 (13.4)	<0.001
Intravenous thrombolytic therapy	1303 (8.6)	912 (8.6)	391 (8.5)	0.705
Discharge medication [*n* (%)]				
Antiplatelet drugs	13,711 (90.4)	9579 (90.8)	4132 (89.4)	0.006
Anticoagulant drugs	449 (3.0)	305 (2.9)	144 (3.1)	0.454
Time interval between stroke onset and MRI scan [h, median (IQR)]	2.0 (1.0–4.0)	2.0 (1.0–4.0)	2.0 (1.0–4.0)	0.010

SD. Standard deviation; BMI. Body mass index; IQR. Interquartile range; MRI. Magnetic resonance imaging; IS. Ischemic stroke

Table S3 Baseline characteristics of the GOLDEN BRIDGE II validation cohort (*n*=14,949)

**Characteristic**	**GOLDEN BRIDGE II validation cohort**
Age (year, mean±SD)	65.9±11.3
Female [*n* (%)]	5369 (35.9)
BMI (kg/m^2, mean±SD)	24.0±3.4
Current smoking [*n* (%)]	4631 (31.0)
History of hypertension [*n* (%)]	9079 (60.7)
Diabetes mellitus	3285 (22.0)
Disorders of lipid metabolism	220 (1.5)
Ischemic stroke	3539 (23.7)
Myocardial infarction	1391 (9.3)
Baseline NIHSS score [score, median (IQR)]	3.0 (1.0–5.0)
Baseline infarct volume [ml, median (IQR)]	1.26 (0.56–4.21)
90-day recurrent ischemic stroke [*n* (%)]	504 (3.4)
90-day all bleeding events [*n* (%)]	154 (1.0)

Baseline infarct volume was converted from mm^3 in the statistical output to ml for presentation. IQR. Interquartile range; NIHSS. National Institutes of Health Stroke Scale; SD. Standard deviation; BMI. Body mass index

Table S4 Associations of infarct volume with recurrent ischemic stroke (IS) and intracerebral hemorrhage (ICH) within restricted NIHSS score ranges

Characteristics	Events/Patients (%)	Unadjusted model		Adjusted model*		Adjusted model†	
		HR (95% CI)	*P*-value	*HR* (95% CI)	*P*-value	*HR* (95% CI)	*P*-value
Recurrent IS							
NIHSS score=0–3							
Per SD of ln-transformed infarct volume	275/5235 (5.25)	1.36 (1.22–1.52)	<0.001	1.39 (1.24–1.55)	<0.001	1.39 (1.25–1.55)	<0.001
NIHSS score=4–5							
Per SD of ln-transformed infarct volume	144/2130 (6.76)	1.33 (1.14–1.55)	<0.001	1.33 (1.14–1.55)	<0.001	1.32 (1.14–1.55)	<0.001
NIHSS score=6–15							
Per SD of ln-transformed infarct volume	188/2916 (6.45)	1.22 (1.06–1.41)	0.005	1.24 (1.07–1.43)	<0.001	1.25 (1.07–1.45)	<0.001
NIHSS score ≥16							
Per SD of ln-transformed infarct volume	24/264 (9.09)	1.39 (0.88–2.22)	0.161	1.53 (0.95–2.47)	0.084	1.53 (0.95–2.47)	0.084
ICH							
NIHSS score=0–3							
Per SD of ln-transformed infarct volume	51/5235 (0.97)	2.73 (2.11–3.54)	<0.001	2.53 (1.94–3.29)	<0.001	2.53 (1.94–3.29)	<0.001
NIHSS score=4–5							
Per SD of ln-transformed infarct volume	25/2130 (1.17)	1.88 (1.33–2.66)	<0.001	1.78 (1.25–2.55)	0.002	1.77 (1.24–2.54)	0.002
NIHSS score=6–15							
Per SD of ln-transformed infarct volume	79/2916 (2.71)	3.16 (2.49–4.01)	<0.001	3.19 (2.40–4.24)	<0.001	2.90 (2.15–3.91)	<0.001
NIHSS score ≥16							
Per SD of ln-transformed infarct volume	29/264 (10.98)	2.55 (1.50–4.33)	<0.001	3.08 (1.58–6.01)	0.001	3.16 (1.61–6.22)	<0.001

Per standard deviation (SD) values of ln-transformed infarct volume were calculated separately for each stratified group of NIHSS score. In patients with an NIHSS score of 0–3, the SD is 1.46; in patients with an NIHSS score of 4–5, the SD is 1.40; in patients with an NIHSS score of 6–15, the SD is 1.64; and in patients with an NIHSS score of ≥16, the SD is 1.82. *Adjusted for age, sex, body mass index, medical history (smoking, history of hypertension, diabetes mellitus, lipid metabolism disorders, ischemic

stroke, myocardial infarction), medication history (antihypertensive drugs, hypoglycemic drugs, lipid-lowering drugs, intravenous thrombolytic therapy), and discharge medication (antiplatelet drugs, anticoagulant drugs). [†]Adjusted for age, sex, body mass index, medical history (smoking, history of hypertension, diabetes mellitus, lipid metabolism disorders, ischemic stroke, myocardial infarction), medication history (antihypertensive drugs, hypoglycemic drugs, lipid-lowering drugs, intravenous thrombolytic therapy), discharge medication (antiplatelet drugs, anticoagulant drugs), and NIHSS score. NIHSS. National institutes of health stroke scale; SD. Standard deviation; ln. logarithmically transformed (base e); *HR*. Hazard ratio; CI. Confidence interval

Table S5 Associations of NIHSS score with recurrent ischemic stroke (IS) and intracerebral hemorrhage (ICH) within restricted infarct volume ranges

**Variable**	**Events/Patients (%)**	**Unadjusted model**		**Adjusted model***		**Adjusted model†**	
		***HR* (95% CI)**	***P*-value**	***HR* (95% CI)**	***P*-value**	***HR* (95% CI)**	***P*-value**
Recurrent IS							
Infarct volume <5.0 ml							
NIHSS score <3 (ref.)	134/3002 (4.46)	1	-	1	-	1	-
NIHSS score ≥3	235/4306 (5.46)	1.23 (0.99–1.52)	0.059	1.17 (0.95–1.45)	0.153	1.08 (0.87–1.34)	0.491
Infarct volume=5.0–9.9 ml							
NIHSS score <4 (ref.)	36/496 (7.26)	1	-	1	-	1	-
NIHSS score ≥4	39/606 (6.44)	0.89 (0.56–1.40)	0.606	0.92 (0.58–1.45)	0.713	0.91 (0.57–1.43)	0.672
Infarct volume=10.0–29.9 ml							
NIHSS score <5 (ref.)	50/579 (8.64)	1	-	1	-	1	-
NIHSS score ≥5	56/660 (8.48)	0.99 (0.67–1.44)	0.941	0.92 (0.62–1.37)	0.694	0.92 (0.62–1.36)	0.670
Infarct volume = 30.0–69.9 ml							
NIHSS score <6 (ref.)	24/267 (8.99)	1	-	1	-	1	-
NIHSS score ≥6	19/286 (6.64)	0.73 (0.40–1.34)	0.309	0.70 (0.37–1.33)	0.281	0.69 (0.36–1.31)	0.256
Infarct volume ≥70.0 ml							
NIHSS score <11 (ref.)	20/159 (12.58)	1	-	1	-	1	-
NIHSS score ≥11	18/184 (9.78)	0.79 (0.42–1.49)	0.465	0.55 (0.27–1.13)	0.105	0.45 (0.21–0.95)	0.037
ICH							
Infarct volume <5.0 ml							
NIHSS score <3 (ref.)	13/3002 (0.43)	1	-	1	-	1	-
NIHSS score ≥3	26/4306 (0.60)	1.51 (0.76–3.00)	0.236	1.16 (0.58–2.31)	0.676	1.10 (0.55–2.22)	0.787

Variable	Events/Patients (%)	Unadjusted model		Adjusted model*		Adjusted model†	
		***HR* (95% CI)**	***P*-value**	***HR* (95% CI)**	***P*-value**	***HR* (95% CI)**	***P*-value**
Infarct volume = 5.0–9.9 ml							
NIHSS score <4 (ref.)	4/496 (0.81)	1	-	1	-	1	-
NIHSS score ≥4	5/606 (0.83)	1.03 (0.28–3.82)	0.970	0.77 (0.19–3.12)	0.714	0.75 (0.18–3.05)	0.685
Infarct volume = 10.0–29.9 ml							
NIHSS score <5 (ref.)	22/579 (3.80)	1	-	1	-	1	-
NIHSS score ≥5	31/660 (4.70)	1.25 (0.72–2.15)	0.432	0.85 (0.46–1.58)	0.613	0.86 (0.46–1.59)	0.629
Infarct volume = 30.0–69.9 ml							
NIHSS score <6 (ref.)	7/267 (2.62)	1	-	1	-	1	-
NIHSS score ≥6	29/286 (10.14)	4.03 (1.76–9.19)	0.001	3.14 (1.27–7.75)	0.013	3.16 (1.28–7.82)	0.013
Infarct volume ≥70.0 ml							
NIHSS score <11 (ref.)	15/159 (9.43)	1	-	1	-	1	-
NIHSS score ≥11	32/184 (17.39)	1.86 (1.01–3.44)	0.047	1.72 (0.84–3.50)	0.136	1.67 (0.80–3.50)	0.174

Median NIHSS scores were calculated separately for each stratified group of infarct volume. NIHSS was dichotomized at the stratum-specific median to maintain balanced comparisons; therefore, the cut points differed across infarct-volume strata. "-" indicates not applicable. *Adjusted for age, sex, body mass index, medical history (smoking, history of hypertension, diabetes mellitus, disorders of lipid metabolism, ischemic stroke, myocardial infarction), medication history (antihypertensive, hypoglycemic, and lipid-lowering drugs, intravenous thrombolytic therapy), medication at discharge (antiplatelet and anticoagulant drugs). †Adjusted for the variables in * plus standardized ln-transformed infarct volume. CI. Confidence interval; *HR*. Hazard ratio; ln. Logarithmically transformed (base e); NIHSS. National Institutes of Health Stroke Scale

Table S6 Associations of infarct volume and NIHSS score with recurrent ischemic stroke (IS) and intracerebral hemorrhage (ICH) in men

**Variable**	**Events/Patients (%)**	**Unadjusted model**		**Adjusted model***		**Adjusted model†/‡**	
		***HR* (95% CI)**	***P*-value**	***HR* (95% CI)**	***P*-value**	***HR* (95% CI)**	***P*-value**
Recurrent IS							
Infarct volume							
Per SD of ln-transformed infarct volume	404/7324 (5.52)	1.26 (1.15–1.39)	<0.001	1.24 (1.13–1.37)	<0.001	1.25 (1.13–1.39)†	<0.001†
NIHSS score							
NIHSS score <4 (ref.)	179/3749 (4.77)	1	-	1	-	1	-
NIHSS score ≥4	225/3575 (6.29)	1.33 (1.09–1.62)	<0.001	1.28 (1.05–1.57)	<0.001	1.16 (0.94–1.42)‡	0.164‡
ICH							
Infarct volume							
Per SD of ln-transformed infarct volume	123/7324 (1.68)	2.91 (2.46–3.45)	<0.001	2.47 (2.07–2.94)	<0.001	2.40 (1.98–2.92)†	<0.001†
NIHSS score							
NIHSS score <4 (ref.)	36/3749 (0.96)	1	-	1	-	1	-
NIHSS score ≥4	87/3575 (2.43)	2.63 (1.78–3.89)	<0.001	2.07 (1.38–3.10)	<0.001	1.20 (0.79–1.84)‡	0.397‡

Standard deviation (SD) values of ln-transformed infarct volume were calculated, with an SD of 1.61, and the median NIHSS score was 4. "-" indicates not applicable. *Adjusted for age, body mass index, medical history (smoking, history of hypertension, diabetes mellitus, disorders of lipid metabolism, ischemic stroke, myocardial infarction), medication history (antihypertensive, hypoglycemic, and lipid-lowering drugs, intravenous thrombolytic therapy), and medication at discharge (antiplatelet and anticoagulant drugs). †Adjusted for the variables in * plus NIHSS score. ‡Adjusted for the variables in * plus standardized ln-transformed infarct volume. CI. Confidence interval; *HR*. Hazard ratio; ln. Logarithmically transformed (base e); NIHSS. National Institutes of Health Stroke Scale; SD. Standard deviation

Table S7 Associations of infarct volume and NIHSS score with recurrent ischemic stroke (IS) and intracerebral hemorrhage (ICH) in women

**Variable**	**Events/Patients (%)**	**Unadjusted model**		**Adjusted model***		**Adjusted model†/‡**	
		***HR* (95% CI)**	***P*-value**	***HR* (95% CI)**	***P*-value**	***HR* (95% CI)**	***P*-value**
Recurrent IS							
Infarct volume							
Per SD of ln-transformed infarct volume	227/3221 (7.05)	1.51 (1.34–1.71)	<0.001	1.51 (1.33–1.71)	<0.001	1.56 (1.36–1.79)†	<0.001†
NIHSS score							
NIHSS score <4 (ref.)	96/1486 (6.46)	1	-	1	-	1	-
NIHSS score ≥4	131/1735 (7.55)	1.18 (0.90–1.53)	0.226	1.10 (0.85–1.45)	0.445	0.90 (0.68–1.19)‡	0.457‡
ICH							
Infarct volume							
Per SD of ln-transformed infarct volume	61/3221 (1.89)	3.64 (2.86–4.62)	<0.001	2.96 (2.32–3.78)	<0.001	2.66 (2.01–3.52)†	<0.001†
NIHSS score							
NIHSS score <4 (ref.)	15/1486 (1.01)	1	-	1	-	1	-
NIHSS score ≥4	46/1735 (2.65)	2.65 (1.48–4.74)	0.001	1.97 (1.09–3.57)	0.025	0.97 (0.51–1.84)‡	0.931‡

Standard deviation (SD) values of ln-transformed infarct volume were calculated, with an SD of 1.61, and the median NIHSS score was 4. "-" indicates not applicable. *Adjusted for age, body mass index, medical history (smoking, history of hypertension, diabetes mellitus, disorders of lipid metabolism, ischemic stroke, myocardial infarction), medication history (antihypertensive, hypoglycemic, and lipid-lowering drugs, intravenous thrombolytic therapy), and medication at discharge (antiplatelet and anticoagulant drugs). †Adjusted for the variables in * plus NIHSS score. ‡Adjusted for the variables in * plus standardized ln-transformed infarct volume. CI. Confidence interval; *HR*. Hazard ratio; ln. Logarithmically transformed (base e); NIHSS. National Institutes of Health Stroke Scale; SD. Standard deviation

Table S8 Associations of infarct volume and NIHSS score with recurrent ischemic stroke (IS) and intracerebral hemorrhage (ICH) in patients aged <65 years

**Variable**	**Events/Patients (%)**	**Unadjusted model**		**Adjusted model***		**Adjusted model†/‡**	
		***HR* (95% CI)**	***P*-value**	***HR* (95% CI)**	***P*-value**	***HR* (95% CI)**	***P*-value**
Recurrent IS							
Infarct volume							
Per SD of ln-transformed infarct	328/6029 (5.44)	1.30 (1.17–1.44)	<0.001	1.30 (1.17–1.44)	<0.001	1.30 (1.16–1.46)†	<0.001†
NIHSS score							
NIHSS score <4 (ref.)	152/3164 (4.8)	1	-	1	-	1	-
NIHSS score ≥4	176/2865 (6.14)	1.29 (1.04–1.60)	0.021	1.22 (0.98–1.52)	0.073	1.09 (0.87–1.36)‡	0.473‡
ICH							
Infarct volume							
Per SD of ln-transformed infarct	86/6029 (1.43)	2.93 (2.39–3.60)	<0.001	2.52 (2.04–3.11)	<0.001	2.49 (1.98–3.12)†	<0.001†
NIHSS score							
NIHSS score <4 (ref.)	27/3164 (0.85)	1	-	1	-	1	-
NIHSS score ≥4	59/2865 (2.06)	2.43 (1.54–3.83)	<0.001	1.89 (1.18–3.02)	0.008	1.15 (0.71–1.88)‡	0.572‡

Standard deviation (SD) values of ln-transformed infarct volume were calculated, with an SD of 1.61, and the median NIHSS score was 4. "-" indicates not applicable. *Adjusted for age, sex, body mass index, medical history (smoking, history of hypertension, diabetes mellitus, disorders of lipid metabolism, ischemic stroke, myocardial infarction), medication history (antihypertensive, hypoglycemic, and lipid-lowering drugs, intravenous thrombolytic therapy), and medication at discharge (antiplatelet and anticoagulant drugs). †Adjusted for the variables in * plus NIHSS score. ‡Adjusted for the variables in * plus standardized ln-transformed infarct volume. CI. Confidence interval; *HR*. Hazard ratio; ln. Logarithmically transformed (base e); NIHSS. National Institutes of Health Stroke Scale; SD. Standard deviation.

Table S9 Associations of infarct volume and NIHSS score with recurrent ischemic stroke (IS) and intracerebral hemorrhage (ICH) in patients aged ≥65 years

**Variable**	**Events/Patients (%)**	**Unadjusted model**		**Adjusted model***		**Adjusted model†/‡**	
		***HR* (95% CI)**	***P*-value**	***HR* (95% CI)**	***P*-value**	***HR* (95% CI)**	***P*-value**
Recurrent IS							
Infarct volume							
Per SD of ln-transformed infarct	303/4516 (6.71)	1.37 (1.24–1.52)	<0.001	1.37 (1.23–1.52)	<0.001	1.40 (1.24–1.57)†	<0.001†
NIHSS score							
NIHSS score <4 (ref.)	123/2071 (5.94)	1	-	1	-	1	-
NIHSS score ≥4	180/2445 (7.36)	1.25 (0.99–1.57)	0.055	1.19 (0.94–1.50)	0.148	1.01 (0.80–1.29)‡	0.908‡
ICH							
Infarct volume							
Per SD of ln-transformed infarct	98/4516 (2.17)	3.25 (2.69–3.91)	<0.001	2.68 (2.20–3.25)	<0.001	2.40 (1.92–3.00)†	<0.001†
NIHSS score							
NIHSS score <4 (ref.)	24/2071 (1.16)	1	-	1	-	1	-
NIHSS score ≥4	74/2445 (3.03)	2.75 (1.72–4.39)	<0.001	2.13 (1.32–3.45)	0.002	1.09 (0.66–1.83)‡	0.729‡

Standard deviation (SD) values of ln-transformed infarct volume were calculated, with an SD of 1.61, and the median NIHSS score was 4. "-" indicates not applicable. *Adjusted for age, sex, body mass index, medical history (smoking, history of hypertension, diabetes mellitus, disorders of lipid metabolism, ischemic stroke, myocardial infarction), medication history (antihypertensive, hypoglycemic, and lipid-lowering drugs, intravenous thrombolytic therapy), and medication at discharge (antiplatelet and anticoagulant drugs). †Adjusted for the variables in * plus NIHSS score. ‡Adjusted for the variables in * plus standardized ln-transformed infarct volume. CI. Confidence interval; *HR*. Hazard ratio; ln. Logarithmically transformed (base e); NIHSS. National Institutes of Health Stroke Scale; SD. Standard deviation

Table S10 Comparative discrimination of NIHSS score and infarct volume for 90-day outcomes

Cohort	Outcome	Events/Patients (%)	Harrell's C-index (95% CI)				ΔC-index (volume-NIHSS)	*P*-value
			Base clinical model	Base+NIHSS	Base+infarct volume	Base+NIHSS+infarct volume		
CNSR-III	Recurrent IS	631/10,545	0.57 (0.54–0.60)	0.57 (0.55–0.60)	0.61 (0.58–0.64)	0.61 (0.58–0.64)	0.04	0.001
	ICH	184/10,545	0.59 (0.54–0.65)	0.71 (0.67–0.75)	0.81 (0.78–0.85)	0.82 (0.78–0.85)	0.11	<0.001
GOLDEN BRIDGE II	Recurrent IS	504/14,949	0.57 (0.54–0.60)	0.57 (0.54–0.60)	0.61 (0.58–0.64)	0.62 (0.59–0.65)	0.04	0.002
	All bleeding events	154/14,949	0.58 (0.52–0.65)	0.63 (0.57–0.68)	0.65 (0.60–0.70)	0.66 (0.61–0.71)	0.03	0.245

The base clinical model included age, sex, body mass index, and medical history (smoking, hypertension, diabetes mellitus, disorders of lipid metabolism, ischemic stroke, and myocardial infarction). Infarct volume was modeled per SD of ln-transformed infarct volume. ΔC-index was calculated as C-index (base+infarct volume) minus C-index (base+NIHSS); positive values indicate better discrimination for infarct volume than NIHSS. CI. Confidence interval; ICH. Intracerebral hemorrhage; NIHSS. National Institutes of Health Stroke Scale

Table S11 Sensitivity analysis on the associations of infarct volume and NIHSS score with recurrent ischemic stroke (IS) and intracerebral hemorrhage (ICH) when adjusted for additional covariates

Variable	Events/Patients (%)	Unadjusted model		Adjusted model*/†		Adjusted model‡/§	
		HR **(95% CI)**	***P*-value**	***HR*** **(95% CI)**	***P*-value**	***HR*** **(95% CI)**	***P*-value**
Recurrent IS							
Infarct volume							
Per SD of ln-transformed infarct	631/10,545 (5.98)	1.34 (1.24–1.44)	<0.001	1.25 (1.14–1.37)	<0.001	1.27 (1.15–1.39)‡	<0.001‡
NIHSS score							
NIHSS score <4 (ref.)	275/5235 (5.25)	1	-	1	-	1	-
NIHSS score ≥4	356/5310 (6.70)	1.29 (1.10–1.51)	0.002	1.05 (0.89–1.24)	0.552	1.02 (0.87–1.21)§	0.793§
ICH							
Infarct volume							
Per SD of ln-transformed infarct	184/10,545 (1.74)	3.13 (2.73–3.59)	<0.001	2.56 (2.15–3.05)	<0.001	2.52 (2.11–2.99)‡	<0.001‡
NIHSS score							
NIHSS score <4 (ref.)	51/5235 (0.97)	1	-	1	-	1	-
NIHSS score ≥4	133/5310 (2.50)	2.64 (1.91–3.66)	<0.001	1.12 (0.79–1.60)	0.534	1.12 (0.78–1.60)§	0.549§

Standard deviation (SD) values of ln-transformed infarct volume were calculated, with an SD of 1.61, and the median NIHSS score was 4. "-" indicates not applicable. *Adjusted for age, sex, body mass index, medical history (smoking, history of hypertension, diabetes mellitus, disorders of lipid metabolism, ischemic stroke, myocardial infarction), medication history (antihypertensive, hypoglycemic, and lipid-lowering drugs, intravenous thrombolytic therapy), medication at discharge (antiplatelet and anticoagulant drugs), NIHSS score, and TOAST etiology. †Adjusted for age, sex, body mass index, medical history (smoking, history of hypertension, diabetes mellitus, disorders of lipid metabolism, ischemic stroke, myocardial infarction), medication history (antihypertensive, hypoglycemic, and lipid-lowering drugs, intravenous thrombolytic therapy), medication at discharge (antiplatelet and anticoagulant drugs), standardized ln-transformed infarct volume, and TOAST etiology. ‡Adjusted for the variables in * plus the time interval between NIHSS assessment and MRI scan. §Adjusted for the variables in † plus the time interval between NIHSS assessment and MRI scan. CI. Confidence interval; *HR*. Hazard ratio; ln. Logarithmically transformed (base e); NIHSS. National Institutes of Health Stroke Scale; SD. Standard deviation; TOAST. Trial of ORG 10172 in Acute Stroke Treatment.

Table S12 Associations of infarct volume and NIHSS score with recurrent ischemic stroke (IS) and intracerebral hemorrhage (ICH) in patients with time from stroke onset to magnetic resonance imaging scan ≤2 d

Variable	Events/Patients (%)	Unadjusted model		Adjusted model*		Adjusted model†/‡	
		***HR* (95% CI)**	***P*-value**	***HR* (95% CI)**	***P*-value**	***HR* (95% CI)**	***P*-value**
Recurrent IS							
Infarct volume							
Per SD of ln-transformed infarct volume	396/5668 (6.99)	1.21 (1.14–1.28)	<0.001	1.21 (1.14–1.28)	<0.001	1.22 (1.14–1.30)†	<0.001†
NIHSS score							
NIHSS score <4 (ref.)	165/2731 (6.04)	1	-	1	-	1	-
NIHSS score ≥4	231/2937 (7.87)	1.31 (1.08–1.61)	0.007	1.27 (1.04–1.56)	0.020	1.10 (0.89–1.35)‡	0.385‡
ICH							
Infarct volume							
Per SD of ln-transformed infarct volume	90/5668 (1.59)	2.84 (2.35–3.43)	<0.001	2.32 (1.91–2.83)	<0.001	2.39 (1.92–2.99)†	<0.001†
NIHSS score							
NIHSS score <4 (ref.)	24/2731 (0.88)	1	-	1	-	1	-
NIHSS score ≥4	66/2937 (2.25)	2.57 (1.61–4.11)	<0.001	1.90 (1.18–3.08)	0.009	1.11 (0.67–1.84)‡	0.692‡

Standard deviation (SD) values of ln-transformed infarct volume were calculated, with an SD of 1.60, and the median NIHSS score was 4. "-" indicates not applicable. *Adjusted for age, sex, body mass index, medical history (smoking, history of hypertension, diabetes mellitus, disorders of lipid metabolism, ischemic stroke, myocardial infarction), medication history (antihypertensive, hypoglycemic, and lipid-lowering drugs, intravenous thrombolytic therapy), and medication at discharge (antiplatelet and anticoagulant drugs). †Adjusted for the variables in * plus NIHSS score. ‡Adjusted for the variables in * plus standardized ln-transformed infarct volume. CI. Confidence interval; *HR*. Hazard ratio; ln. Logarithmically transformed (base e); NIHSS. National Institutes of Health Stroke Scale; SD. Standard deviation.

Table S13 Associations of infarct volume and NIHSS score with ischemic stroke (IS) and intracerebral hemorrhage (ICH) in patients with time from stroke onset to magnetic resonance imaging scan ≥3 d

**Variable**	**Events/Patients (%)**	**Unadjusted model**		**Adjusted model***		**Adjusted model†/‡**	
		***HR* (95% CI)**	***P*-value**	***HR* (95% CI)**	***P*-value**	***HR* (95% CI)**	***P*-value**
Recurrent IS							
Infarct volume							
Per SD of ln-transformed infarct	235/5668 (4.15)	1.20 (1.12–1.30)	<0.001	1.20 (1.11–1.30)	<0.001	1.22 (1.13–1.33)†	<0.001†
NIHSS score							
NIHSS score <4 (ref.)	85/1841 (4.62)	1	-	1	-	1	-
NIHSS score ≥4	150/3020 (4.97)	1.08 (0.83–1.41)	0.575	0.97 (0.74–1.28)	0.846	0.85 (0.64–1.12)‡	0.236‡
ICH							
Infarct volume							
Per SD of ln-transformed infarct	94/5668 (1.66)	3.48 (2.84–4.27)	<0.001	2.99 (2.42–3.69)	<0.001	2.59 (2.05–3.26)†	<0.001†
NIHSS score							
NIHSS score <4 (ref.)	19/1841 (1.03)	1	-	1	-	1	-
NIHSS score ≥4	75/3020 (2.48)	2.56 (1.53–4.28)	<0.001	2.02 (1.19–3.41)	0.009	1.11 (0.64–1.92)‡	0.706‡

Standard deviation (SD) values of ln-transformed infarct volume were calculated, with an SD of 1.61, and the median NIHSS score was 3. "-" indicates not applicable. *Adjusted for age, sex, body mass index, medical history (smoking, history of hypertension, diabetes mellitus, disorders of lipid metabolism, ischemic stroke, myocardial infarction), medication history (antihypertensive, hypoglycemic, and lipid-lowering drugs, intravenous thrombolytic therapy), and medication at discharge (antiplatelet and anticoagulant drugs). †Adjusted for the variables in * plus NIHSS score. ‡Adjusted for the variables in * plus standardized ln-transformed infarct volume. CI. Confidence interval; *HR*. Hazard ratio; ln. Logarithmically transformed (base e); NIHSS. National Institutes of Health Stroke Scale; SD. Standard deviation.

Table S14 Associations of infarct volume with hemorrhagic transformation

Infarct volume (ml)	Hemorrhagic transformation	
	Events/Patients (%)	*HR* (95% CI)
<1 (Ref.)	6/3426 (0.18)	1
1–2	6/2037 (0.29)	1.68 (0.54–5.22)
2–3	6/921 (0.65)	3.73 (1.20–11.56)
3–4	4/517 (0.77)	4.43 (1.25–15.71)
4–5	1/407 (0.25)	1.40 (0.17–11.66)
5–6	2/275 (0.73)	4.16 (0.84–20.62)
6–7	1/262 (0.38)	2.18 (0.26–18.10)
7–8	1/206 (0.49)	2.78 (0.33–23.05)
8–9	1/185 (0.54)	3.09 (0.37–25.63)
9–10	2/174 (1.15)	6.60 (1.33–32.71)
10–11	4/139 (2.88)	16.68 (4.71–59.12)
11–12	2/113 (1.77)	10.19 (2.06–50.47)
12–13	1/99 (1.01)	5.81 (0.70–48.29)
13–14	1/85 (1.18)	6.73 (0.81–55.94)
14–15	2/77 (2.60)	15.07 (3.04–74.64)
15–16	3/95 (3.16)	18.18 (4.55–72.68)
16–17	1/54 (1.85)	10.62 (1.28–88.22)
17–18	0/60 (0.00)	NA
18–19	6/66 (9.09)	53.91 (17.39–167.16)
19–20	1/47 (2.13)	12.20 (1.47–101.36)
20–21	2/49 (4.08)	23.64 (4.77–117.14)
21–22	3/53 (5.66)	32.40 (8.10–129.53)
22–23	3/52 (5.77)	33.16 (8.29–132.58)
23–24	3/41 (7.32)	43.12 (10.78–172.41)
24–25	2/43 (4.65)	26.86 (5.42–133.06)
25–26	4/37 (10.81)	64.42 (18.18–228.27)
26–27	3/40 (7.50)	43.76 (10.94–174.98)
27–28	1/30 (3.33)	19.31 (2.32–160.36)
28–29	1/37 (2.70)	15.61 (1.88–129.64)
29–30	3/22 (13.64)	83.90 (20.98–335.50)

Adjusted for age, sex, body mass index, medical history (smoking, history of hypertension, diabetes mellitus, disorders of lipid metabolism, ischemic stroke, myocardial infarction), medication history (antihypertensive, hypoglycemic, and lipid-lowering drugs, intravenous thrombolytic therapy), and medication at discharge (antiplatelet and anticoagulant drugs). *HR*. Hazard ratio; CI. Confidence interval

Table S15 Associations of infarct volume with primary intracerebral hemorrhage

Infarct volume (ml)	Primary intracerebral hemorrhage	
	**Events/Patients (%)**	***HR* (95% CI)**
<1 (Ref.)	8/3418 (0.23)	1
1–2	7/2030 (0.34)	1.68 (0.59–4.80)
2–3	0/921 (0)	0 (0–NA)
3–4	1/516 (0.19)	0.95 (0.12–7.69)
4–5	1/406 (0.25)	1.20 (0.15–9.78)
5–6	1/274 (0.36)	1.78 (0.22–14.47)
6–7	1/261 (0.38)	1.87 (0.23–15.17)
7–8	0/206 (0)	0 (0–NA)
8–9	0/185 (0)	0 (0–NA)
9–10	1/173 (0.57)	2.83 (0.35–22.96)
10–11	0/139 (0)	0 (0–NA)
11–12	1/112 (0.88)	4.35 (0.54–35.38)
12–13	2/97 (2.02)	9.97 (2.07–48.00)
13–14	1/84 (1.18)	5.81 (0.71–47.19)
14–15	0/77 (0)	0 (0–NA)
15–16	2/93 (2.11)	10.46 (2.17–50.35)
16–17	0/54 (0)	0 (0–NA)
17–18	0/60 (0)	0 (0–NA)
18–19	0/66 (0)	0 (0–NA)
19–20	1/46 (2.13)	10.45 (1.29–84.95)
20–21	0/49 (0)	0 (0–NA)
21–22	0/53 (0.55)	0 (0–NA)
22–23	0/52 (0)	0 (0–NA)
23–24	0/41 (0)	0 (0–NA)
24–25	0/43 (0)	0 (0–NA)
25–26	1/36 (2.70)	13.54 (1.67–110.07)
26–27	1/39 (2.50)	12.47 (1.53–101.35)
27–28	0/30 (0)	0 (0–NA)
28–29	0/37 (0)	0 (0–NA)
29–30	0/22 (0)	0 (0–NA)

Adjusted for age, sex, body mass index, medical history (smoking, history of hypertension, diabetes mellitus, disorders of lipid metabolism, ischemic stroke, myocardial infarction), medication history (antihypertensive, hypoglycemic, and lipid-lowering drugs, intravenous thrombolytic therapy), and medication at discharge (antiplatelet and anticoagulant drugs). *HR*. Hazard ratio; CI. Confidence interval

Table S16 Influence of infarct topology of single infarction versus multiple infarctions on recurrent ischemic stroke (IS) and intracerebral hemorrhage (ICH)

**Variable**	**Events/Patients (%)**	**Unadjusted model**		**Adjusted model***		**Adjusted model†**	
		***HR* (95% CI)**	***P*-value**	***HR* (95% CI)**	***P*-value**	***HR* (95% CI)**	***P*-value**
Recurrent IS							
Single infarction (ref.)	221/4793 (4.61)	1	-	1	-	1	-
Multiple infarction	368/5240 (7.02)	1.54 (1.30–1.82)	<0.001	1.49 (1.26–1.76)	<0.001	1.12 (0.92–1.37)	0.257†
ICH							
Single infarction (ref.)	36/4793 (0.75)	1	-	1	-	1	-
Multiple infarction	140/5240 (2.67)	3.63 (2.51–5.24)	<0.001	2.91 (2.00–4.24)	<0.001	0.80 (0.51–1.25)	0.325†

"-" indicates not applicable. *Adjusted for age, sex, body mass index, medical history (smoking, history of hypertension, diabetes mellitus, lipid metabolism disorders, ischemic stroke, myocardial infarction), medication history (antihypertensive drugs, hypoglycemic drugs, lipid-lowering drugs, intravenous thrombolytic therapy), discharge medication (antiplatelet drugs, anticoagulant drugs). †Adjusted for the variables in * plus ln-transformed infarct volume. *HR*. Hazard ratio; CI. Confidence interval

Table S17 Influence of the infarct topology of cortex involved versus not involved infarction on recurrent ischemic stroke (IS) and intracerebral hemorrhage (ICH)

**Variable**	**Events/Patients (%)**	**Unadjusted model**		**Adjusted model***		**Adjusted model†**	
		***HR* (95% CI)**	***P*-value**	***HR* (95% CI)**	***P*-value**	***HR* (95% CI)**	***P*-value**
Recurrent IS							
Cortex not infarction (ref.)	411/7414 (5.54)	1	-	1	-	1	-
Cortex involved infarction	220/3131 (7.03)	1.27 (1.08–1.50)	0.004	1.24 (1.05–1.46)	0.013	0.85 (0.70–1.04)	0.107†
ICH							
Cortex not infarction (ref.)	80/7414 (1.08)	1	-	1	-	1	-
Cortex involved infarction	104/3131 (3.32)	3.15 (2.35–4.23)	<0.001	2.51 (1.85–3.41)	<0.001	0.78 (0.55–1.12)	0.176†

"-" indicates not applicable. *Adjusted for age, sex, body mass index, medical history (smoking, history of hypertension, diabetes mellitus, lipid metabolism disorders, ischemic stroke, myocardial infarction), medication history (antihypertensive drugs, hypoglycemic drugs, lipid-lowering drugs, intravenous thrombolytic therapy), discharge medication (antiplatelet drugs, anticoagulant drugs). †Adjusted for the variables in * plus ln-transformed infarct volume. *HR*. Hazard ratio; CI. Confidence interval

Table S18 Influence of the infarct topology of anterior circulation involved infarction versus posterior circulation involved infarction on recurrent ischemic stroke (IS) and intracerebral hemorrhage (ICH)

**Variable**	**Events/Patients (%)**	**Unadjusted model**		**Adjusted model***		**Adjusted model†**	
		***HR* (95% CI)**	***P*-value**	***HR* (95% CI)**	***P*-value**	***HR* (95% CI)**	***P*-value**
Recurrent IS							
Infarction related to posterior circulation (ref.)	194/3299 (5.88)	1	-	1	-	1	-
Infarction related to anterior circulation	370/6518 (5.68)	0.97 (0.81–1.15)	0.693	0.98 (0.82–1.16)	0.779	0.82 (0.68–0.98)	0.032
Infarction related to anterior and posterior circulation	67/728 (9.20)	1.60 (1.21–2.11)	0.001	1.51 (1.14–2.00)	0.004	1.15 (0.86–1.54)	0.336
ICH							
Infarction related to posterior circulation (ref.)	23/3299 (0.70)	1	-	1	-	1	-
Infarction related to anterior circulation	142/6518 (2.18)	3.17 (2.04–4.94)	<0.001	2.69 (1.72–4.22)	<0.001	1.43 (0.90–2.27)	0.131
Infarction related to anterior and posterior circulation	19/728 (2.61)	3.82 (2.07–7.05)	<0.001	2.86 (1.52–5.35)	0.001	1.18 (0.61–2.26)	0.622

"-" indicates not applicable. *Adjusted for age, sex, body mass index, medical history (smoking, history of hypertension, diabetes mellitus, lipid metabolism disorders, ischemic stroke, myocardial infarction), medication history (antihypertensive drugs, hypoglycemic drugs, lipid-lowering drugs, intravenous thrombolytic therapy), discharge medication (antiplatelet drugs, anticoagulant drugs). †Adjusted for the variables in * plus ln-transformed infarct volume. *HR*. Hazard ratio; CI. Confidence interval

Table S19 Associations of infarct volume and NIHSS score with 1-year risks of recurrent ischemic stroke (IS) and intracerebral hemorrhage (ICH)

Variable	Events/Patients (%)	Unadjusted model		Adjusted model*		Adjusted model†/‡	
		***HR* (95% CI)**	***P*-value**	***HR* (95% CI)**	***P*-value**	***HR* (95% CI)**	***P*-value**
Recurrent IS							
Infarct volume							
Per SD of ln-transformed infarct volume	962/10,545 (9.12)	1.28 (1.20–1.36)	<0.001	1.28 (1.21–1.36)	<0.001	1.29 (1.21–1.38)†	<0.001†
NIHSS score							
NIHSS score <4 (ref.)	418/5235 (7.98)	1	-	1	-	1	-
NIHSS score ≥4	544/5310 (10.24)	1.31 (1.15–1.49)	<0.001	1.23 (1.08–1.40)	0.002	1.10 (0.96–1.26)‡	0.170‡
ICH							
Infarct volume							
Per SD of ln-transformed infarct volume	212/10,545 (2.01)	2.60 (2.29–2.94)	<0.001	2.25 (1.98–2.56)	<0.001	2.07 (1.79–2.39)†	<0.001†
NIHSS score							
NIHSS score <4 (ref.)	61/5235 (1.17)	1	-	1	-	1	-
NIHSS score ≥4	151/5310 (2.84)	2.45 (1.82–3.29)	<0.001	1.97 (1.45–2.67)	<0.001	1.23 (0.90–1.70)‡	0.201‡

Standard deviation (SD) values of ln-transformed infarct volume were calculated, with an SD of 1.61, and the median NIHSS score was 4. "-" indicates not applicable. *Adjusted for age, sex, body mass index, medical history (smoking, history of hypertension, diabetes mellitus, disorders of lipid metabolism, ischemic stroke, myocardial infarction), medication history (antihypertensive, hypoglycemic, and lipid-lowering drugs, intravenous thrombolytic therapy), and medication at discharge (antiplatelet and anticoagulant drugs). †Adjusted for the variables in * plus NIHSS score. ‡Adjusted for the variables in * plus standardized ln-transformed infarct volume. CI. Confidence interval; *HR*. Hazard ratio; ln. Logarithmically transformed (base e); NIHSS. National Institutes of Health Stroke Scale; SD. Standard deviation.

Table S20 Associations of infarct volume and NIHSS score with 5-year risks of recurrent ischemic stroke (IS) and intracerebral hemorrhage (ICH)

**Variable**	**Events/Patients (%)**	**Unadjusted model**		**Adjusted model***		**Adjusted model†/‡**	
		***HR* (95% CI)**	***P*-value**	***HR* (95% CI)**	***P*-value**	***HR* (95% CI)**	***P*-value**
Recurrent IS							
Infarct volume							
Per SD of ln-transformed infarct volume	1560/10,545 (14.79)	1.19 (1.14–1.25)	<0.001	1.22 (1.16–1.28)	<0.001	1.20 (1.14–1.27)†	<0.001†
NIHSS score							
NIHSS score <4 (ref.)	676/5235 (12.91)	1	-	1	-	1	-
NIHSS score ≥4	884/5310 (16.65)	1.35 (1.22–1.49)	<0.001	1.28 (1.16–1.42)	<0.001	1.18 (1.06–1.31)‡	0.002‡
ICH							
Infarct volume							
Per SD of ln-transformed infarct volume	254/10,545 (2.41)	2.41 (2.14–2.72)	<0.001	2.10 (1.86–2.38)	<0.001	1.95 (1.70–2.24)†	<0.001†
NIHSS score							
NIHSS score <4 (ref.)	83/5235 (1.59)	1	-	1	-	1	-
NIHSS score ≥4	171/5310 (3.22)	2.09 (1.58–2.76)	<0.001	1.69 (1.27–2.25)	<0.001	1.09 (0.81–1.48)‡	0.563‡

Standard deviation (SD) values of ln-transformed infarct volume were calculated, with an SD of 1.61, and the median NIHSS score was 4. "-" indicates not applicable. *Adjusted for age, sex, body mass index, medical history (smoking, history of hypertension, diabetes mellitus, disorders of lipid metabolism, ischemic stroke, myocardial infarction), medication history (antihypertensive, hypoglycemic, and lipid-lowering drugs, intravenous thrombolytic therapy), and medication at discharge (antiplatelet and anticoagulant drugs). †Adjusted for the variables in * plus NIHSS score. ‡Adjusted for the variables in * plus standardized ln-transformed infarct volume. CI. Confidence interval; *HR*. Hazard ratio; ln. Logarithmically transformed (base e); NIHSS. National Institutes of Health Stroke Scale; SD. Standard deviation

Table S21 Sensitivity analysis on the associations of infarct volume and NIHSS score with recurrent ischemic stroke (IS) and intracerebral hemorrhage (ICH) after excluding patients with prior stroke

Variable	Events/Patients (%)	Unadjusted model		Adjusted model*		Adjusted model†/‡	
		***HR* (95% CI)**	***P*-value**	***HR* (95% CI)**	***P*-value**	***HR* (95% CI)**	***P*-value**
Recurrent IS							
Infarct volume							
Per SD of ln-transformed infarct volume	455/8282 (5.49)	1.31 (1.20–1.43)	<0.001	1.28 (1.17–1.40)	<0.001	1.31 (1.19–1.45)†	<0.001†
NIHSS score							
NIHSS score <4 (ref.)	209/4217 (4.96)	1	-	1	-	1	-
NIHSS score ≥4	246/4065 (6.05)	1.23 (1.02–1.48)	0.028	1.16 (0.96–1.40)	0.129	1.02 (0.84–1.24)‡	0.868‡
ICH							
Infarct volume							
Per SD of ln-transformed infarct volume	455/8282 (5.49)	1.31 (1.20–1.43)	<0.001	1.28 (1.17–1.40)	<0.001	1.31 (1.19–1.45)†	<0.001†
NIHSS score							
NIHSS score <4 (ref.)	38/4217 (0.90)	1	-	1	-	1	-
NIHSS score ≥4	104/4065 (2.56)	2.94 (2.02–4.28)	<0.001	2.21 (1.50–3.25)	<0.001	1.18 (0.78–1.77)‡	0.434‡

Standard deviation (SD) values of ln-transformed infarct volume were calculated, with an SD of 1.61, and the median NIHSS score was 4. "-" indicates not applicable. *Adjusted for age, sex, body mass index, medical history (smoking, history of hypertension, diabetes mellitus, disorders of lipid metabolism, myocardial infarction), medication history (antihypertensive, hypoglycemic, and lipid-lowering drugs, intravenous thrombolytic therapy), medication at discharge (antiplatelet and anticoagulant drugs). †Adjusted for the variables in * plus NIHSS score. ‡Adjusted for the variables in * plus standardized ln-transformed infarct volume. CI. Confidence interval; *HR*. Hazard ratio; ln. Logarithmically transformed (base e); NIHSS. National Institutes of Health Stroke Scale; SD. Standard deviation.

Table S22 Sensitivity analysis on the associations of infarct volume and NIHSS score with recurrent ischemic stroke (IS) and intracerebral hemorrhage (ICH) after additional exclusion of patients with pre-stroke antithrombotic therapy (antiplatelet or anticoagulant)

**Variable**	**Events/Patients (%)**	**Unadjusted model**		**Adjusted model***		**Adjusted model†/‡**	
		***HR* (95% CI)**	***P*-value**	***HR* (95% CI)**	***P*-value**	***HR* (95% CI)**	***P*-value**
Recurrent IS							
Infarct volume							
Per SD of ln-transformed infarct volume	406/7491 (5.42)	1.30 (1.19–1.43)	<0.001	1.29 (1.18–1.42)	<0.001	1.33 (1.20–1.48)†	<0.001†
NIHSS score							
NIHSS score <4 (ref.)	186/3822 (4.87)	1	-	1	-	1	-
NIHSS score ≥4	220/3669 (6.00)	1.24 (1.02–1.51)	0.030	1.19 (0.97–1.45)	0.092	1.04 (0.84–1.28)‡	0.721‡
ICH							
Infarct volume							
Per SD of ln-transformed infarct volume	132/7491 (1.76)	3.45 (2.91–4.08)	<0.001	2.93 (2.46–3.50)	<0.001	2.71 (2.23–3.30)†	<0.001†
NIHSS score							
NIHSS score <4 (ref.)	35/3822 (0.92)	1	-	1	-	1	-
NIHSS score ≥4	97/3669 (2.64)	3.00 (2.03–4.43)	<0.001	2.27 (1.52–3.39)	<0.001	1.14 (0.74–1.75)‡	0.542‡

Standard deviation (SD) values of ln-transformed infarct volume were calculated, with an SD of 1.61, and the median NIHSS score was 4. "-" indicates not applicable. *Adjusted for age, sex, body mass index, medical history (smoking, history of hypertension, diabetes mellitus, disorders of lipid metabolism, myocardial infarction), medication history (antihypertensive, hypoglycemic, and lipid-lowering drugs, intravenous thrombolytic therapy), medication at discharge (antiplatelet and anticoagulant drugs). †Adjusted for the variables in * plus NIHSS score. ‡Adjusted for the variables in * plus standardized ln-transformed infarct volume. CI. Confidence interval; *HR*. Hazard ratio; ln. Logarithmically transformed (base e); NIHSS. National Institutes of Health Stroke Scale; SD. Standard deviation

Table S23 Sensitivity analysis on the associations of infarct volume and NIHSS score with recurrent ischemic stroke (IS) and intracerebral hemorrhage (ICH) after additional exclusion of patients with prior cardiovascular disease, hepatic or renal impairment, or pre-existing disability (mRS ≥2)

Variable	Events/Patients (%)	Unadjusted model		Adjusted model*		Adjusted model†/‡	
		***HR* (95% CI)**	***P*-value**	***HR* (95% CI)**	***P*-value**	***HR* (95% CI)**	***P*-value**
Recurrent IS							
Infarct volume							
Per SD of ln-transformed infarct volume	341/6287 (5.42)	1.36 (1.22–1.50)	<0.001	1.34 (1.21–1.49)	<0.001	1.37 (1.22–1.54)†	<0.001†
NIHSS score							
NIHSS score <4 (ref.)	160/3322 (4.82)	1	-	1	-	1	-
NIHSS score ≥4	181/2965 (6.10)	1.28 (1.03–1.58)	0.024	1.22 (0.99–1.52)	0.066	1.06 (0.85–1.33)‡	0.597‡
ICH							
Infarct volume							
Per SD of ln-transformed infarct volume	99/6287 (1.57)	3.35 (2.75–4.08)	<0.001	2.77 (2.26–3.39)	<0.001	2.60 (2.08–3.26)†	<0.001†
NIHSS score							
NIHSS score <4 (ref.)	28/3322 (0.84)	1	-	1	-	1	-
NIHSS score ≥4	71/2965 (2.39)	2.97 (1.91–4.62)	<0.001	2.14 (1.35–3.38)	0.001	1.15 (0.71–1.86)‡	0.581‡

Standard deviation (SD) values of ln-transformed infarct volume were calculated, with an SD of 1.61, and the median NIHSS score was 4. "-" indicates not applicable. *Adjusted for age, sex, body mass index, medical history (smoking, history of hypertension, diabetes mellitus, disorders of lipid metabolism), medication history (antihypertensive, hypoglycemic, and lipid-lowering drugs, intravenous thrombolytic therapy), medication at discharge (antiplatelet and anticoagulant drugs). †Adjusted for the variables in * plus NIHSS score. ‡Adjusted for the variables in * plus standardized ln-transformed infarct volume. CI. Confidence interval; *HR*. Hazard ratio; ln. Logarithmically transformed (base e); NIHSS. National Institutes of Health Stroke Scale; SD. Standard deviation

Table S24 Sensitivity analysis on the associations of infarct volume and NIHSS score with recurrent ischemic stroke (IS) and intracerebral hemorrhage (ICH) after additional exclusion of patients undergoing reperfusion therapy

Variable	Events/Patients (%)	Unadjusted model		Adjusted model*		Adjusted model†/‡	
		***HR* (95% CI)**	***P*-value**	***HR* (95% CI)**	***P*-value**	***HR* (95% CI)**	***P*-value**
Recurrent IS							
Infarct volume							
Per SD of ln-transformed infarct volume	306/5610 (5.45)	1.38 (1.24–1.54)	<0.001	1.37 (1.23–1.53)	<0.001	1.39 (1.23–1.57)†	<0.001†
NIHSS score							
NIHSS score <4 (ref.)	150/3124 (4.80)	1	-	1	-	1	-
NIHSS score ≥4	156/2486 (6.28)	1.32 (1.05–1.65)	0.016	1.25 (1.00–1.57)	0.053	1.08 (0.86–1.37)‡	0.508‡
ICH							
Infarct volume							
Per SD of ln-transformed infarct volume	70/5610 (1.25)	3.74 (2.95–4.75)	<0.001	3.13 (2.47–3.98)	<0.001	2.83 (2.17–3.70)†	<0.001†
NIHSS score							
NIHSS score <4 (ref.)	24/3124 (0.77)	1	-	1	-	1	-
NIHSS score ≥4	46/2486 (1.85)	2.53 (1.53–4.17)	<0.001	2.07 (1.24–3.44)	0.005	1.02 (0.59–1.76)‡	0.946‡

Standard deviation (SD) values of ln-transformed infarct volume were calculated, with an SD of 1.61, and the median NIHSS score was 4. "-" indicates not applicable. *Adjusted for age, sex, body mass index, medical history (smoking, history of hypertension, diabetes mellitus, disorders of lipid metabolism), medication history (antihypertensive, hypoglycemic, and lipid-lowering drugs), medication at discharge (antiplatelet and anticoagulant drugs). †Adjusted for the variables in * plus NIHSS score. ‡Adjusted for the variables in * plus standardized ln-transformed infarct volume. CI. Confidence interval; *HR*. Hazard ratio; ln. Logarithmically transformed (base e); NIHSS. National Institutes of Health Stroke Scale; SD. Standard deviation.

Table S25 Sensitivity analysis on the competing-risk analysis (death as a competing event): associations of infarct volume and NIHSS score with recurrent ischemic stroke (IS) and intracerebral hemorrhage (ICH)

**Variable**	**Events/Patients (%)**	**Unadjusted model**		**Adjusted model***		**Adjusted model†/‡**	
		***SHR* (95% CI)**	***P*-value**	***SHR* (95% CI)**	***P*-value**	***SHR* (95% CI)**	***P*-value**
Recurrent IS							
Infarct volume							
Per SD of ln-transformed infarct volume	631/10,545 (5.98)	1.33 (1.24–1.43)	<0.001	1.32 (1.23–1.42)*	<0.001*	1.34 (1.24–1.46)†	<0.001†
NIHSS score							
NIHSS score <4 (ref.)	275/5235 (5.25)	1	-	1	-	1	-
NIHSS score ≥4	356/5310 (6.70)	1.29 (1.10–1.50)	0.002	1.21 (1.03–1.42)*	0.021*	1.05 (0.89–1.24)‡	0.552‡
ICH							
Infarct volume							
Per SD of ln-transformed infarct volume	184/10,545 (1.74)	3.08 (2.66–3.55)	<0.001	2.59 (2.20–3.04)*	<0.001*	2.45 (2.06–2.91)†	<0.001†
NIHSS score							
NIHSS score <4 (ref.)	51/5235 (0.97)	1	-	1	-	1	-
NIHSS score ≥4	133/5310 (2.50)	2.58 (1.87–3.57)	<0.001	1.97 (1.42–2.74)*	<0.001*	1.10 (0.77–1.55)‡	0.608‡

Standard deviation (SD) values of ln-transformed infarct volume were calculated, with an SD of 1.61, and the median NIHSS score was 4. "**-**" indicates not applicable. *Adjusted for age, sex, body mass index, medical history (smoking, history of hypertension, diabetes mellitus, disorders of lipid metabolism, ischemic stroke, myocardial infarction), medication history (antihypertensive, hypoglycemic, and lipid-lowering drugs, intravenous thrombolytic therapy), medication at discharge (antiplatelet and anticoagulant drugs). †Adjusted for the variables in * plus NIHSS score. ‡Adjusted for the variables in * plus standardized ln-transformed infarct volume. CI. Confidence interval; *SHR*. Subdistribution hazard ratio; ln. Logarithmically transformed (base e); NIHSS. National Institutes of Health Stroke Scale; SD. Standard deviation

Table S26 External validation of clinically interpretable infarct-volume categories in GOLDEN BRIDGE II

Variable	Events/Patients (%)	Unadjusted model		Adjusted model*		Adjusted model†	
		***HR* (95% CI)**	***P*-value**	***HR* (95% CI)**	***P*-value**	***HR* (95% CI)**	***P*-value**
Recurrent IS							
Infarct volume category							
<9.0 ml (ref.)	398/12,649 (3.15)	1	-	1	-	1	-
9.0–17.9 ml	44/1006 (4.37)	1.40 (1.03–1.92)	0.033	1.40 (1.03–1.91)	0.035	1.44 (1.06–1.97)	0.022
≥18.0 ml	62/1294 (4.79)	1.54 (1.18–2.02)	0.002	1.55 (1.19–2.03)	0.001	1.67 (1.27–2.20)	<0.001
All bleeding events							
Infarct volume category							
<9.0 ml (ref.)	98/12,649 (0.77)	1	-	1	-	1	-
9.0–17.9 ml	20/1006 (1.99)	2.59 (1.60–4.19)	<0.001	2.56 (1.58–4.14)	<0.001	2.36 (1.45–3.83)	<0.001
≥18.0 ml	36/1294 (2.78)	3.64 (2.49–5.34)	<0.001	3.63 (2.47–5.33)	<0.001	2.97 (1.98–4.47)	<0.001

*Adjusted for age, sex, body mass index, and medical history (smoking, hypertension, diabetes mellitus, disorders of lipid metabolism, ischemic stroke, and myocardial infarction). †Adjusted for the variables in * plus NIHSS score. The infarct-volume categories were evaluated as exploratory, clinically interpretable categories and should not be interpreted as validated clinical cutoffs. "-" indicates not applicable. CI. Confidence interval; *HR*. Hazard ratio; NIHSS. National Institutes of Health Stroke Scale; IS. Ischemic stroke

Table S27 Exploratory 1-ml interval event rates and hazard ratios (*HR*s) of ischemic stroke (IS) in GOLDEN BRIDGE II

Infarct volume (ml)	90-day recurrent IS	
	**Event rate (%)**	**Adjusted *HR* (95% CI)***
<1 (Ref.)	2.28	1
1–2	3.68	1.64 (1.27–2.11)
2–3	4.64	2.09 (1.52–2.86)
3–4	4.63	2.04 (1.41–2.97)
4–5	3.50	1.55 (0.94–2.56)
5–6	5.12	2.21 (1.34–3.65)
6–7	6.38	2.89 (1.77–4.72)
7–8	1.17	0.50 (0.19–1.55)
8–9	3.66	1.59 (0.74–3.38)
9–10	7.78	3.49 (1.98–6.16)
10–11	2.08	0.90 (0.29–2.82)
11–12	6.04	2.70 (1.38–5.29)
12–13	4.31	1.88 (0.77–4.59)
13–14	4.76	2.20 (0.90–5.36)
14–15	1.18	0.52 (0.07–3.68)
15–16	2.06	0.93 (0.23–3.75)
16–17	3.70	1.70 (0.54–5.32)
17–18	4.84	2.11 (0.67–6.63)
18–19	4.29	1.84 (0.59–5.78)
19–20	8.77	3.99 (1.63–9.74)
20–21	4.55	2.05 (0.51–8.28)
21–22	1.96	0.94 (0.13–6.74)
22–23	0.00	NE
23–24	3.64	1.60 (0.40–6.47)
24–25	6.82	3.15 (1.00–9.90)
25–26	4.88	2.20 (0.54–8.87)
26–27	17.78	8.94 (4.38–18.25)
27–28	0.00	NE
28–29	0.00	NE
29–30	0.00	NE

Infarct-volume intervals are shown in ml after conversion from mm^3 in the statistical output (1000 mm^3=1.0 ml). Volume-group analyses included 14,189 participants with nonmissing 1-ml interval data. *Adjusted for age, sex, body mass index, and medical history (smoking, hypertension, diabetes mellitus, disorders of lipid metabolism, ischemic stroke, and myocardial infarction). NE indicates not estimable because of zero events in the corresponding infarct-volume interval. CI. Confidence interval; *HR*. Hazard ratio; NE. Not estimable

Table S28 Exploratory 1-ml interval event rates and hazard ratios (*HR*) of bleeding events in GOLDEN BRIDGE II

Infarct volume (ml)	90-day all bleeding events	
	**Event rate (%)**	**Adjusted *HR* (95% CI)***
< 1 (Ref.)	0.65	1
1–2	0.83	1.29 (0.78–2.15)
2–3	0.96	1.52 (0.78–2.95)
3–4	0.95	1.48 (0.66–3.29)
4–5	0.82	1.30 (0.47–3.62)
5–6	0.60	0.92 (0.22–3.82)
6–7	1.06	1.64 (0.51–5.31)
7–8	1.56	2.37 (0.85–6.61)
8–9	1.05	1.56 (0.38–6.47)
9–10	2.40	3.61 (1.29–10.07)
10–11	0.69	1.00 (0.14–7.28)
11–12	0.67	1.04 (0.14–7.55)
12–13	2.59	4.09 (1.27–13.21)
13–14	6.67	10.74 (4.81–23.94)
14–15	0.00	NE
15–16	1.03	1.61 (0.22–11.70)
16–17	0.00	NE
17–18	4.84	7.38 (2.28–23.88)
18–19	0.00	NE
19–20	0.00	NE
20–21	2.27	3.69 (0.51–26.89)
21–22	1.96	3.10 (0.43–22.60)
22–23	6.06	9.66 (2.33–39.96)
23–24	1.82	2.91 (0.40–21.21)
24–25	4.55	7.01 (1.69–29.05)
25–26	2.44	3.64 (0.50–26.47)
26–27	2.22	3.43 (0.47–24.95)
27–28	6.25	10.25 (2.47–42.53)
28–29	9.68	15.63 (4.83–50.57)
29–30	0.00	NE

Infarct-volume intervals are shown in ml after conversion from mm^3 in the statistical output (1000 mm^3=1.0 ml). Volume-group analyses included 14,189 participants with nonmissing 1-ml interval data. *Adjusted for age, sex, body mass index, and medical history (smoking, hypertension, diabetes mellitus, disorders of lipid metabolism, ischemic stroke, and myocardial infarction). NE indicates not estimable because of zero events in the corresponding infarct-volume interval. CI. Confidence interval; *HR*. Hazard ratio; NE. Not estimable

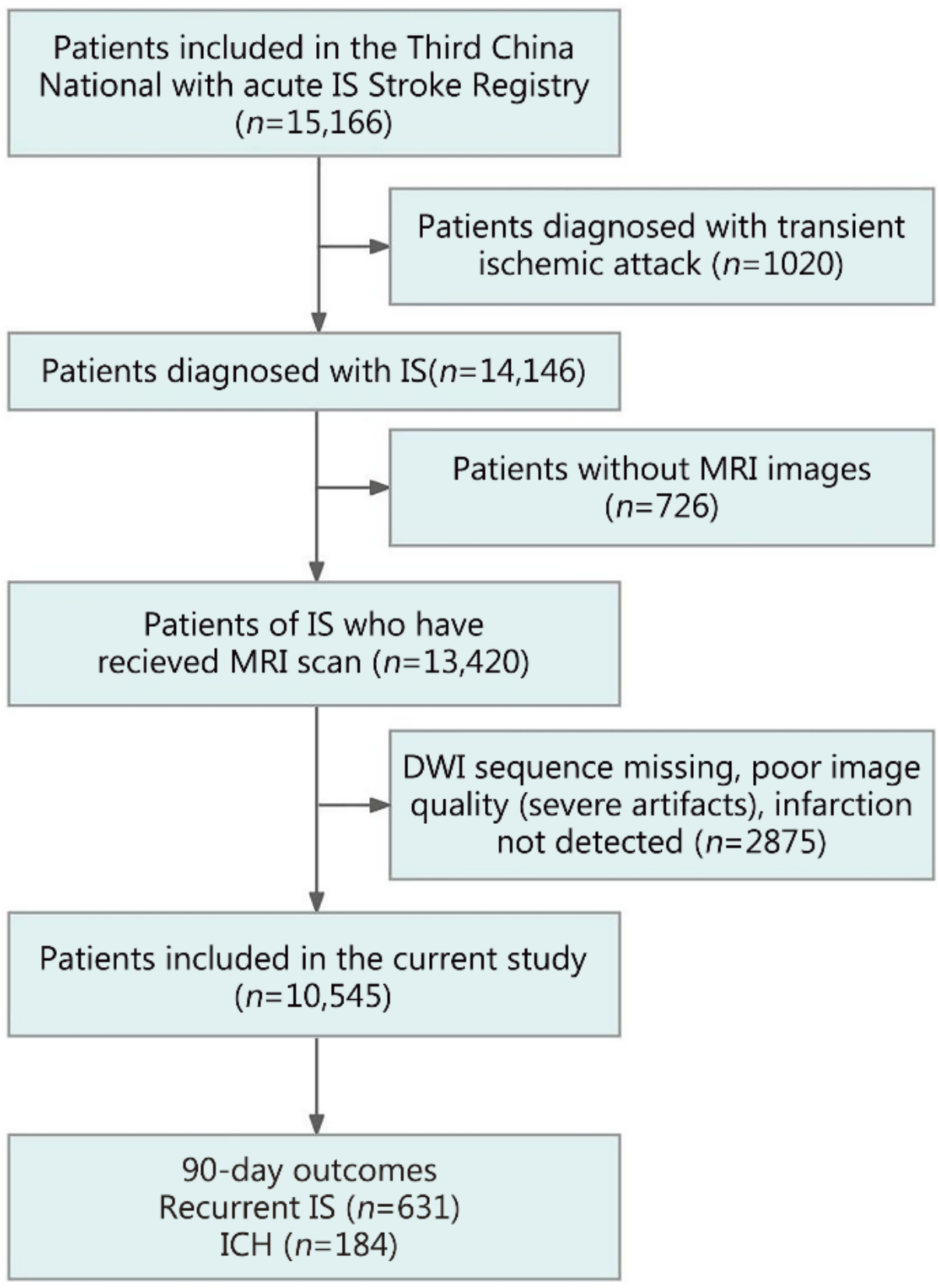

Fig. S1 Study flow diagram. Of 15,166 registered patients, 10,545 were included in the primary analysis; 631 experienced recurrent IS and 184 experienced ICH within 90 d. MRI. Magnetic resonance imaging; DWI. Diffusion-weighted imaging; IS. Ischemic stroke; ICH. Intracerebral hemorrhage

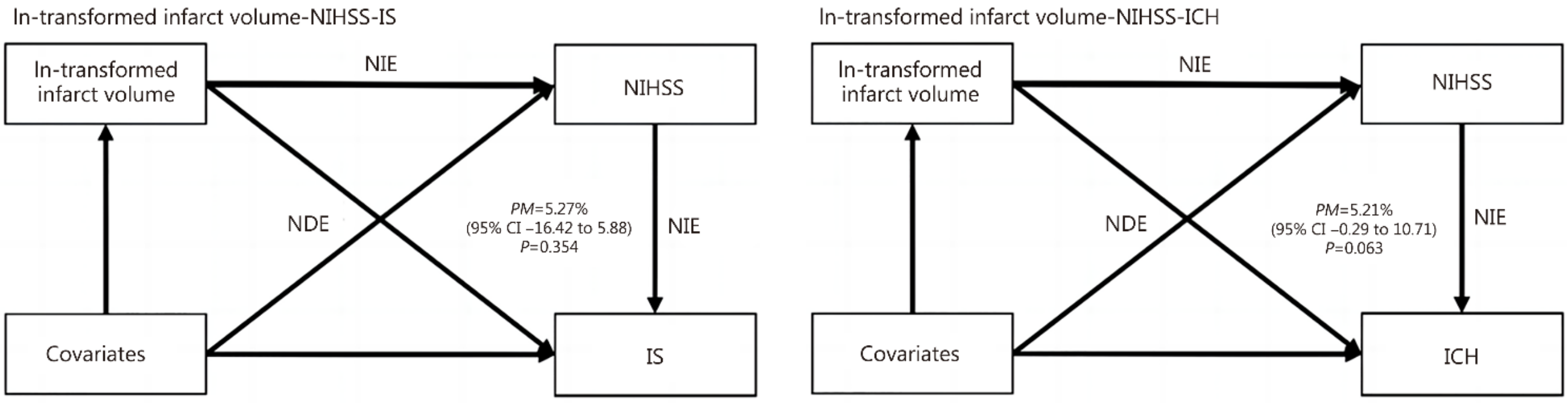

Effect	*OR* (95% CI)	*P*-value
TE	1.20(1.14–1.26)	<0.001
NDE	1.21(1.15–1.28)	<0.001
NIE	0.99(0.97–1.01)	0.348

Effect	*OR* (95% CI)	*P*-value
TE	1.84(1.66–2.01)	<0.001
NDE	1.80(1.61–1.98)	<0.001
NIE	1.02(1.00–1.05)	0.062

Fig. S2 Mediation effect of NIHSS score in the association between infarct volume and primary study outcomes. Models were adjusted for age, sex, body mass index, medical history (smoking, history of hypertension, diabetes mellitus, disorders of lipid metabolism, ischemic stroke, and myocardial infarction), medication history (antihypertensive, hypoglycemic, and lipid-lowering drugs, and intravenous thrombolytic therapy), and discharge medications (antiplatelet and anticoagulant drugs). TE. Total effect; NDE. Natural direct effect; NIE. Natural indirect effect; *PM*. Proportion mediated; NIHSS. National Institutes of Health Stroke Scale; IS. Ischemic stroke; ICH. Intracerebral hemorrhage; *OR*. Odds ratio; CI. Confidence interval

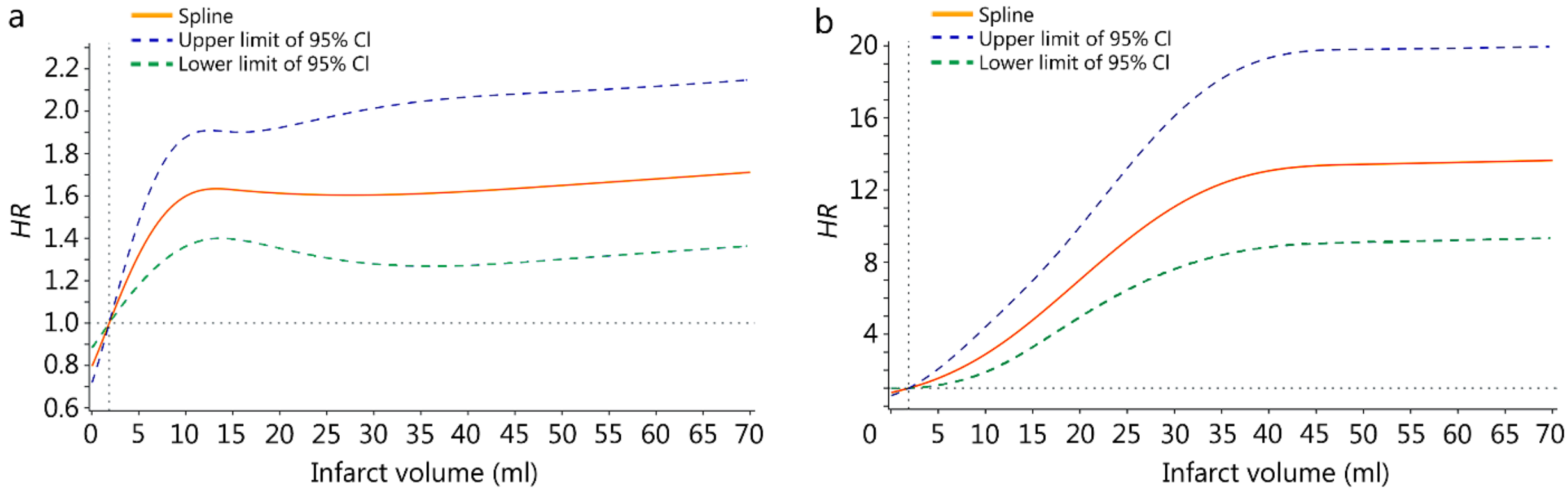

Fig. S3 Trends in infarct volume (0–70 ml), ischemic stroke, and intracerebral hemorrhage (*n*=10,202). The association of infarct volume-ischemic stroke (**a**) and infarct volume-intracerebral hemorrhage (**b**) on a continuous scale with restricted cubic spline curves. Orange central lines represent the estimated hazard ratios (*HR*s) for ischemic stroke and intracerebral hemorrhage, respectively, with blue dotted lines denoting 95% CI. The vertical dotted lines indicate the median infarct volume selected as the reference, with *HR* = 1. Infarct volume is shown in the range of 0–70 ml. Adjusted for age, sex, body mass index, medical history (smoking, history of hypertension, diabetes mellitus, lipid metabolism disorders, ischemic stroke, myocardial infarction), medication history (antihypertensive drugs, hypoglycemic drugs, lipid-lowering drugs, intravenous thrombolytic therapy), discharge medication (antiplatelet drugs, anticoagulant drugs). CI. Confidence interval

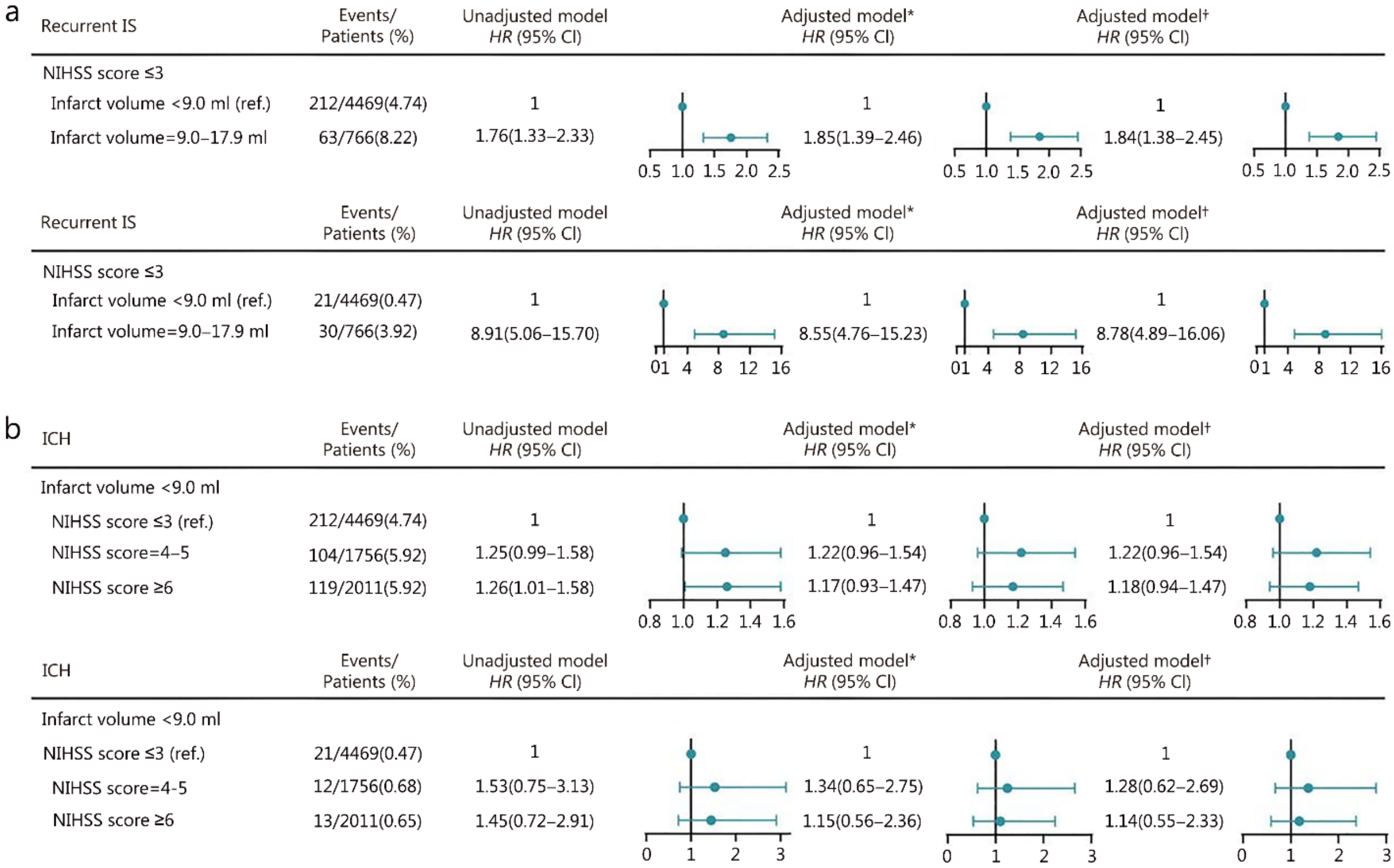

Fig. S4 Risk consistency in "infarct volume <9 ml" versus "NIHSS score ≤3" for recurrent ischemic stroke (**a**) (*n*=5235) and intracerebral hemorrhage (**b**) (*n*=8236). *Adjusted for age, sex, body mass index, medical history (smoking, hypertension, diabetes mellitus, disorders of lipid metabolism, ischemic stroke, and myocardial infarction), medication history (antihypertensive, hypoglycemic, and lipid-lowering drugs, intravenous thrombolytic therapy), and medication at discharge (antiplatelet and anticoagulant drugs). †The bootstrap-corrected *HR* and 95% CI were based on 1000 independent bootstrap runs. CI. Confidence interval; *HR*. hazard ratio; NIHSS. National Institutes of Health Stroke Scale; ref. Reference

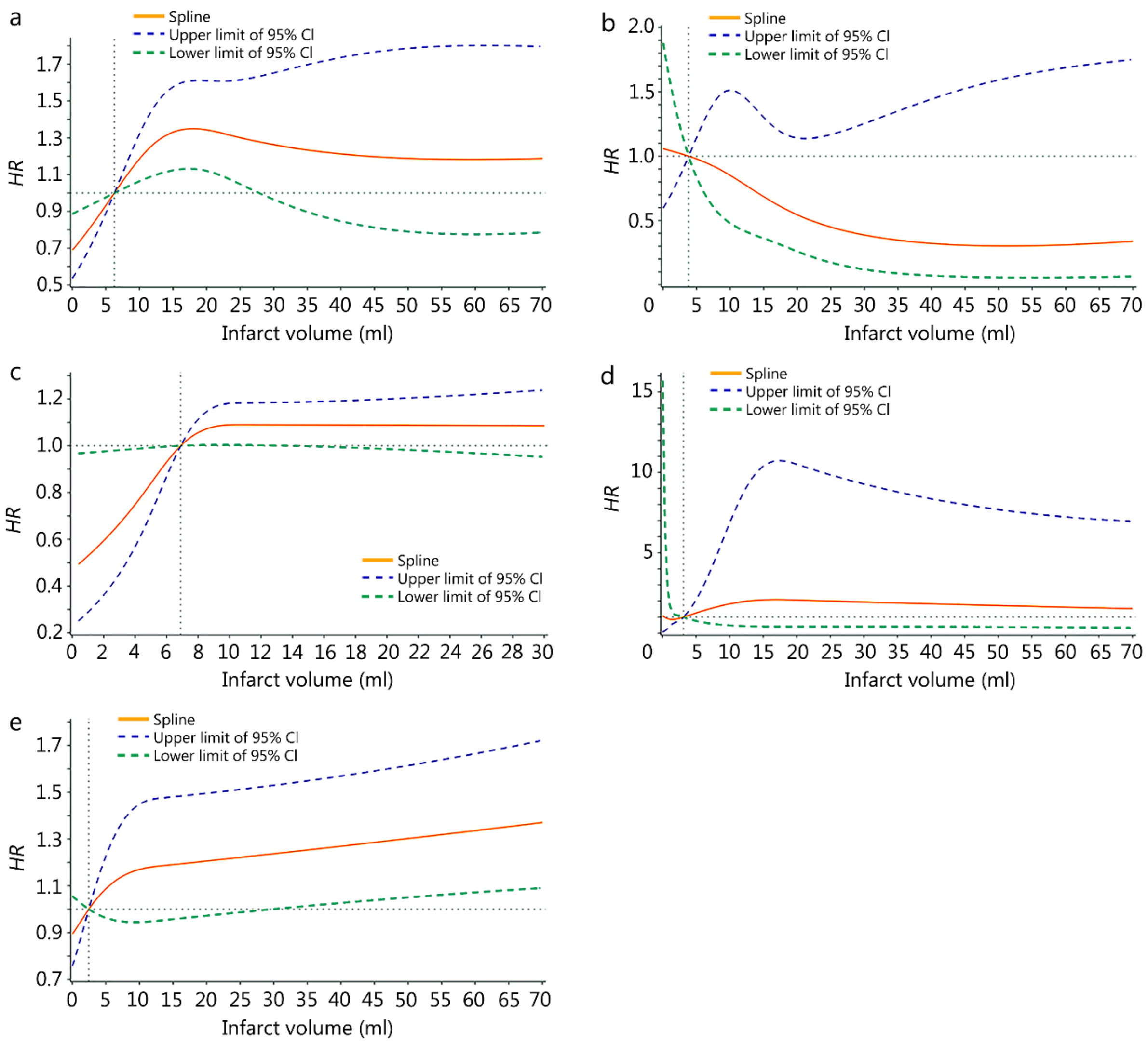

Fig. S5 Different trends in infarct volume (0–70 ml) and risk of ischemic stroke according to TOAST classifications. The association of infarct volume-ischemic stroke on a continuous scale with restricted cubic spline curves. Orange central lines represent the estimated hazard ratios (*HR*s) for ischemic stroke, with blue dotted lines denoting 95% CI. The vertical dotted lines indicate the median infarct volume selected as the reference, with *HR*=1. Infarct volume is shown in the range of 0–70 ml. **a** Trends in infarct volume and risk of ischemic stroke in large-artery atherosclerosis etiology (*n*=2706). **b** Trends in infarct volume and risk of ischemic stroke in cardioembolism etiology (*n*=604). **c** Trends in infarct volume and risk of ischemic stroke in small-artery occlusion etiology (*n*=2690). **d** Trends in infarct volume and risk of ischemic stroke in other determined etiology (*n*=135). **e** Trends in infarct volume and risk of ischemic stroke in undetermined etiology (*n*=4067). Adjusted for age, sex, body mass index, medical history (smoking, history of hypertension, diabetes mellitus, lipid metabolism disorders, ischemic stroke, myocardial infarction), medication history (antihypertensive drugs, hypoglycemic drugs, lipid-lowering drugs, intravenous thrombolytic therapy), discharge medication (antiplatelet drugs, anticoagulant drugs). TOAST. The Trial of ORG 10,172 in acute stroke treatment. CI. Confidence interval

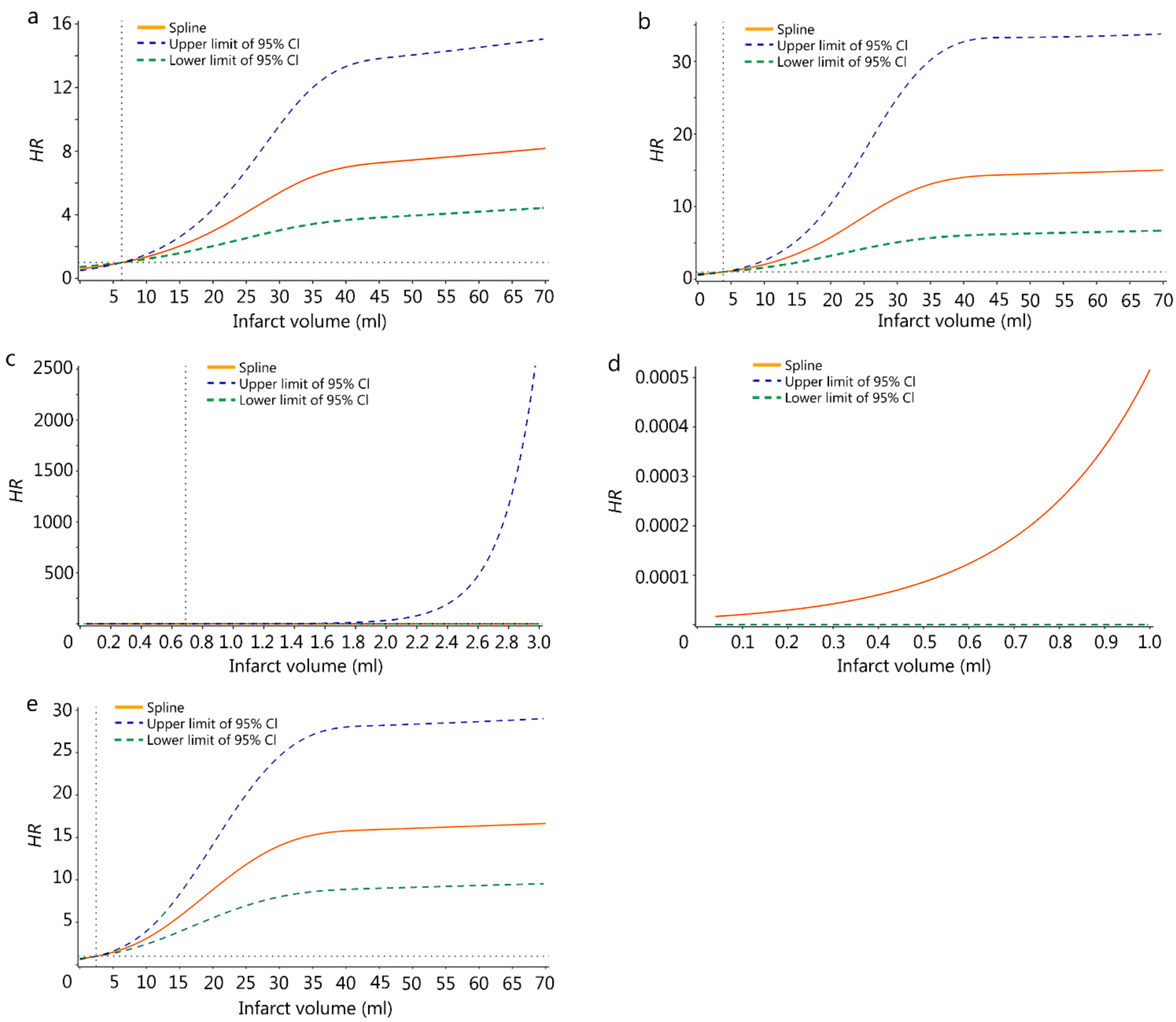

Fig. S6 Different trends in infarct volume (0–70 ml) and risk of intracerebral hemorrhage according to TOAST classifications. The association of infarct volume-intracerebral hemorrhage on a continuous scale with restricted cubic spline curves. Orange central lines represent the estimated hazard ratios for intracerebral hemorrhage, with blue dotted lines denoting 95% CI. The vertical dotted lines indicate the median infarct volume selected as the reference, with hazard ratio (*HR*) = 1. Infarct volume is shown in the range of 0–70 ml. **a** Trends in infarct volume and risk of intracerebral hemorrhage in large-artery atherosclerosis etiology (*n*=2706). **b** Trends in infarct volume and risk of intracerebral hemorrhage in cardioembolism etiology (*n*=604). **c** Trends in infarct volume and risk of intracerebral hemorrhage in small-artery occlusion etiology (*n*=2690). **d** Trends in infarct volume and risk of intracerebral hemorrhage in other determined etiology (*n*=135). **e** Trends in infarct volume and risk of intracerebral hemorrhage in undetermined etiology (*n*=4067). Adjusted for age, sex, body mass index, medical history (smoking, history of hypertension, diabetes mellitus, lipid metabolism disorders, ischemic stroke, myocardial infarction), medication history (antihypertensive drugs, hypoglycemic drugs, lipid-lowering drugs, intravenous thrombolytic therapy), discharge medication (antiplatelet drugs, anticoagulant drugs). TOAST. the Trial of ORG 10172 in acute stroke treatment; CI. Confidence interval

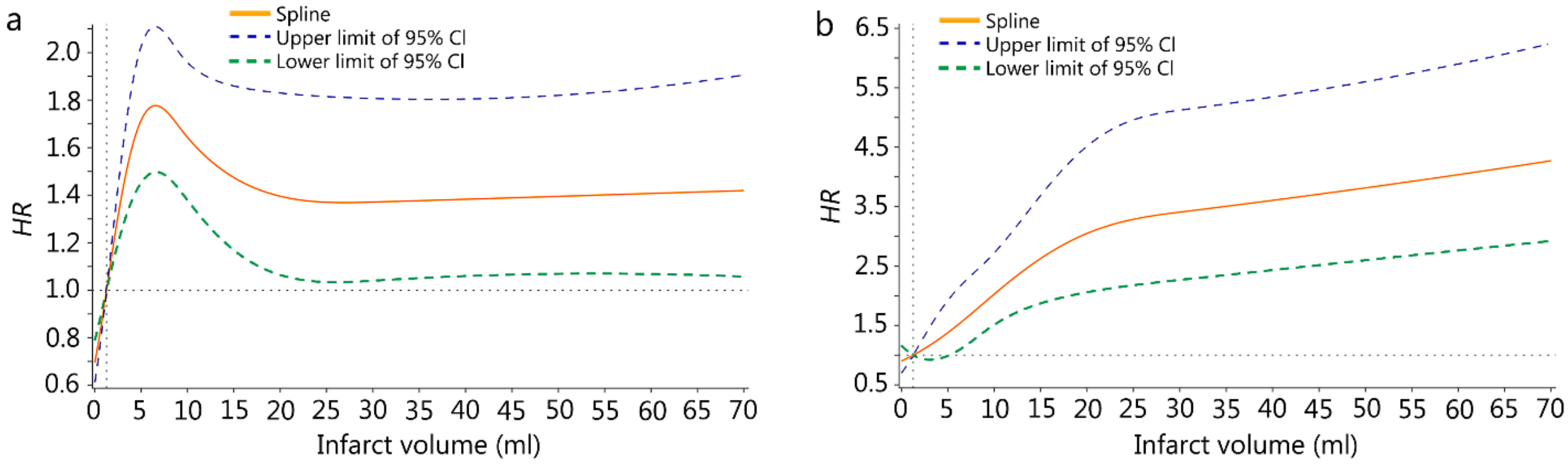

Fig. S7 Restricted cubic spline associations of infarct volume with 90-day recurrent ischemic stroke and all bleeding events in GOLDEN BRIDGE II (*n*=14,949). The associations of infarct volume with 90-day recurrent ischemic stroke (**a**) and 90-day all bleeding events (**b**) on a continuous scale using restricted cubic spline curves in GOLDEN BRIDGE II. Orange central lines represent the estimated hazard ratios (*HR*s), and blue dashed lines denote 95% CIs. The vertical dotted line indicates the median infarct volume selected as the reference, and the horizontal dotted line denotes *HR*=1. Infarct volume is shown in the range of 0–70 ml. Models were adjusted for age, sex, body mass index, and medical history (smoking, hypertension, diabetes mellitus, disorders of lipid metabolism, ischemic stroke, and myocardial infarction). These exploratory curves were used to illustrate dose–response patterns and were not used to define or validate a clinical cutoff. CI. Confidence interval
